# *GFRAL* genetic variations and circulating GFRAL in hyperemesis gravidarum

**DOI:** 10.1016/j.isci.2026.117975

**Published:** 2026-10-23

**Authors:** Beatrice Haack, Zainab H. Neemuchwala, Summer DeVera, Kimber W. MacGibbon, Birgitt Fischer, Florian Höschle, Peter A. Fasching, Andrew Housholder, Eszter Vanky, Manuel A. Friese, Marie Bixo, Louise Stiernman, Emelie Ekwurtzel, Malin Nording, Marie-Therese Vinnars, Jörg Wischhusen, Marlena S. Fejzo

**Affiliations:** 1 Department of Obstetrics and Gynecology, University Hospital Würzburg, Würzburg, Germany; 2 Department of Population and Public Health Sciences, Keck School of Medicine, University of Southern California, Los Angeles, CA, USA; 3 Hyperemesis Education and Research Foundation, 10117 SE Sunnyside Rd, Suite F8, Clackamas, OR 97015, USA; 4 Department of Gynecology and Obstetrics, University Hospital Erlangen, Erlangen, Germany; 5 Morning Sickness Clinic, Birmingham, AL, USA; 6 Department of Clinical and Molecular Medicine, Faculty of Medicine and Health Sciences, Norwegian University of Science and Technology, Trondheim, Norway; 7 Department of Obstetrics and Gynecology, St. Olav’s Hospital, Trondheim University Hospital, Trondheim, Norway; 8 Institute of Neuroimmunology and Multiple Sclerosis, University Medical Center Hamburg-Eppendorf, Hamburg, Germany; 9 Hamburg Center for Translational Immunology, University Medical Center Hamburg-Eppendorf, Hamburg, Germany; 10 Department of Clinical Sciences, Obstetrics and Gynecology, Umeå University, Umeå, Sweden; 11 IVIRMA Global Research Alliance, Livio Umeå, Umeå, Sweden; 12 Department of Chemistry, Umeå University, Umeå, Sweden

**Keywords:** hyperemesis gravidarum, nausea, vomiting, pregnancy, GFRAL, sGFRAL, GDF 15

## Abstract

Hyperemesis gravidarum, the most severe form of nausea and vomiting in pregnancy, is linked to variants in *GDF15* and its receptor *GFRAL*. This study combined exome and targeted sequencing, genotyping, immunoassays, and clinical data to characterize *GFRAL* sequence variation and circulating soluble GFRAL (sGFRAL) in pregnancy. Rare *GFRAL* variants were identified in patients with hyperemesis gravidarum and in asymptomatic pregnancies. A common variant was associated with symptoms persisting beyond 27 weeks’ gestation. sGFRAL was frequently detectable in early pregnancy, with dynamic changes across gestation and inverse correlation with weight loss, and subsequently more common in hyperemesis gravidarum. *In silico* promoter analysis suggested progesterone-dependent repression of *GFRAL* transcription, concordant with sGFRAL downregulation in controls after week 6. In contrast, women with hyperemesis gravidarum maintained soluble receptor levels. These findings implicate genetic and post-translational regulation of GFRAL in pregnancy sickness and support further evaluation of sGFRAL as a biomarker and mechanistic readout.

## Highlights

• Soluble (cleaved) GFRAL is detectable in pregnancy and shows dynamic changes across gestation
• Hyperemesis gravidarum differs from normal pregnancy in soluble GFRAL detection and levels
• *GFRAL* variants in HG cases, African ancestry cases, prolonged symptoms, and symptom-free pregnancies
• Progesterone-dependent GFRAL repression may contribute to pregnancy-related desensitization

## Graphical abstract

**Figure:**
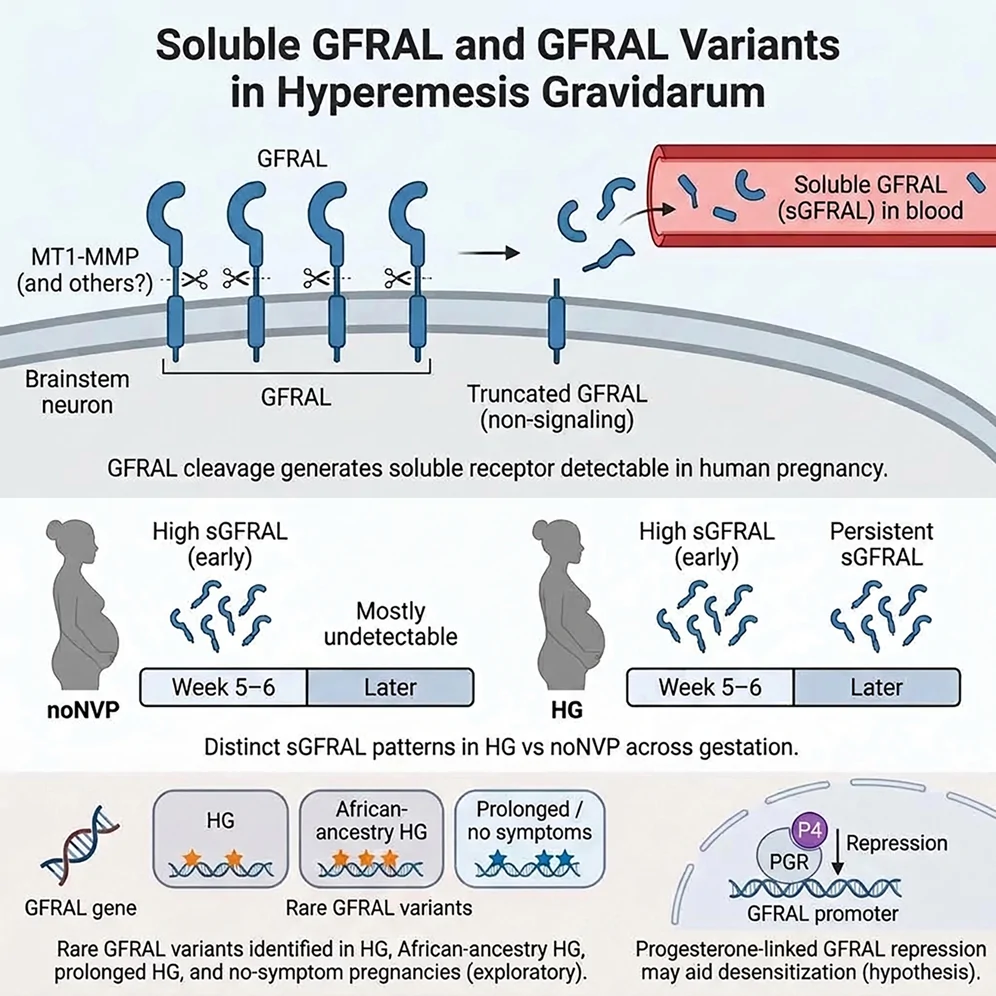

## Teaser

Female reproductive endocrinology

## Introduction

Nausea and vomiting in pregnancy (NVP) affect approximately 70% of pregnancies. The most severe form, Hyperemesis Gravidarum (HG), affects 0.3%–10.8% of pregnancies.^1^ It can be life-threatening and is associated with adverse outcomes. It begins early in pregnancy, affects daily activity with an inability to eat and drink normally, leading to dehydration, undernutrition, and weight loss. HG is the second-leading cause of pregnancy-related hospitalization after preterm birth, yet no FDA-approved medications exist. Recent genetic studies identified variants in growth/differentiation factor 15 (*GDF15*) to be associated with HG.^1^ GDF-15 is a stress-inducible divergent member of the TGF-β superfamily that may play manifold roles in appetite, nausea, metabolic rewiring, placental growth, and immune tolerance during pregnancy.^2^^,^^3^ Its highest physiological expression is in placenta,^4^^,^^5^ where GDF-15 can cause nausea and vomiting by activating glial cell-derived neurotrophic factor receptor alpha-like (GFRAL)^6^^,^^7^^,^^8^^,^^9^ in the brainstem’s nausea and vomiting center. GFRAL is a glycosylphosphatidylinositol-anchored receptor whose expression is essentially restricted to neurons of the *area postrema*, and nucleus of the *tractus solitarius*, where it forms a signaling complex with the RET tyrosine kinase to transduce GDF-15 signals. Proteolytic cleavage at a juxtamembrane site by MT1-MMP/MMP14 releases a soluble ectodomain (sGFRAL), leaving behind a truncated receptor that can no longer be activated by GDF-15.^10^ This could provide a mechanism to dynamically modulate sensitivity to GDF-15. How GFRAL expression and cleavage are regulated in pregnancy is currently unknown. Beyond HG, the pathway is associated with neuroimmune cross-talk,^11^^,^^12^ and with cachexia and chemotherapy-induced nausea and vomiting.^7^^,^^13^ Pfizer completed a phase 2 study of its anti-GDF-15 antibody Ponsegromab, which safely reduced cachexia symptoms while increasing weight and activity in cachexia patients.^14^ Cancer patients with high GDF-15 levels likewise showed increased body weight upon GDF-15 blockade with CatalYm's visugromab.^15^^,^^16^ If proven safe in pregnancy, neutralizing GDF-15 might also be an effective treatment for HG. Alternatively, blocking GFRAL should be beneficial for patients with nausea/vomiting. This might even have the advantage that—still debated—GFRAL-independent local or immune functions of GDF-15 would not be affected.^3^

Risk-associated genetic *GDF15* variants are linked to lower baseline levels of circulating GDF-15 when not pregnant.^17^ Pre-conditioning with normal levels prior to pregnancy may thus result in desensitization, whereas low basal levels correlate with high sensitivity to the rise of GDF-15 during pregnancy. But *GDF15* is not the only gene associated with increased risk. Common variants in the gene coding for its brainstem-restricted receptor, *GFRAL*, and variants in the progesterone receptor (*PGR*) are also associated with the condition.^18^ Little is known about the genetics and molecular mechanism for GFRAL with respect to NVP. C-terminal cleavage at R333-K of human GFRAL followed by shedding by MT1-MMP/MMP14 has been described *in vitro*.^10^ In mice, MT1-MMP haploinsufficiency or inhibition enhances GDF-15’s anorexigenic effects, which confirms GFRAL inactivation by said cleavage. Therefore, the objective of this study was to analyze the nausea and vomiting hormone receptor gene *GFRAL* and soluble (shed) GFRAL in unaffected and NVP-affected controls, and HG cases.

## Results

### Rare and novel *GFRAL* variants selectively detected in HG cases

Whole-exome sequencing of 926 HG cases and 660 controls identified 14 rare *GFRAL* variants occurring exclusively in HG cases, including five missense variants (one novel; three predicted damaging) (Table 1). A case requiring parenteral nutrition carried the p.Met370Thr variant at the GFRAL-RET51 interface, predicted to interfere with activated signaling complex dissociation.^19^ We further identified eight rare intron variants (one novel). A rare 5’ UTR variant (rs138536707) was identified with significantly elevated frequency in African American HG cases (2/32 cases in this, 3/144 in the morning sickness clinic [MSC] cohort; OR = 2.6, 95% CI = 1.05–6.49; *p* = 0.04).^20^

**Table 1:** Rare *GFRAL* variants identified by whole-exome sequencing of 926 HG cases and 660 unaffected controls

Variant type	rs ID number	Chromosome 6 position	Ref base	Alt base	severity^a^ and ancestry	Nucleotide change	Amino acid change	N	Sift prediction	Global allele frequency
**HG, NOT FOUND IN CONTROLS**										

5_prime_UTR_variant	rs138536707	55327553	G	T	AFR, AFR	c.-2G>T	–	2	–	0.00192853
missense_variant	NOVEL	55351490	A	C	EUR	c.608A>C	p.Glu203Ala	1	damaging	–
missense_variant	rs116829590	55351433	A	G	EUR	c.551A>G	p.Gln184Arg	1	damaging	0.0006424
missense_variant	rs145926895	55399429	T	C	AMR, HT AFR	c.1109T>C	p.Met370Thr	2	damaging	0.00033499
missense_variant	rs143766097	55359070	C	A	EUR	c.884C>A	p.Thr295Asn	1	tolerated	0.00016782
missense_variant	rs150000020	55351435	A	C	EUR, EUR	c.553A>C	p.Met185Leu	2	tolerated	0.00018157
intron_variant	NOVEL	55359202	T	A	EUR	c.952 + 64T>A	–	1	–	–
intron_variant	rs148831204	55327595	T	A	EUR	c.22 + 19T>A	–	1	–	2.09E−05
intron_variant	rs759927826	55331654	A	G	EUR	c.23-61A>G	–	1	–	9.08E−05
intron_variant	rs565369124	55333689	T	C	EUR	c.158-97T>C	–	1	–	0.00048162
intron_variant	rs202116103	55351608	C	T	EUR	c.701 + 25C>T	–	1	–	0.00023044
intron_variant	rs1321193778	55351680	C	T	EAS	c.701 + 97C>T	–	1	–	1.40E−05
intron_variant	rs999458640	55359165	TCTA	–	EUR	c.952 + 45_952 + 48del	–	1	–	0.00019037
intron_variant	rs367638155	55359178	C	T	EAS	c.952 + 40C>T	–	1	–	5.76E−05

**CONTROLS, NOT FOUND IN HG CASES**										

frameshift_elongation	rs527905870	55399239	–	A	CA EUR, CA EUR	c.1015dup	p.Ile339AsnfsTer36	2	–	0.00111029
missense_variant	rs377644506	55351478	A	G	EUR	c.596A>G	p.Gln199Arg	1	tolerated	2.79E−05
missense_variant	rs200268366	55351534	C	G	CA EUR, CA EUR	c.652C>G	p.Pro218Ala	2	tolerated	0.0003981
missense_variant	rs962296758	55351547	G	A	EUR	c.665G>A	p.Ser222Asn	1	tolerated	3.49E−05
missense_variant	rs199980249	55351556	G	A	EUR	c.674G>A	p.Arg225His	1	tolerated	4.89E−05
missense_variant	rs148997298	55358952	G	A	EUR	c.766G>A	p.Glu256Lys	1	tolerated	6.29E−05
missense_variant	NOVEL	55399377	A	G	EUR	c.1057A>G	p.Ile353Val	1	tolerated	–
intron_variant	NOVEL	55350188	C	G	EAS	c.370 + 43C>G	–	1	–	–
intron_variant	NOVEL	55359194	T	A	EUR	c.952 + 56T>A	–	1	–	–
intron_variant	rs372846440	55401774	C	T	EUR	c.1122-16C>T	–	1	–	0.00011868
intron_variant	rs374634382	55359176	A	G	EUR	c.952 + 38A>G	–	1	–	0.00040666

A, adenine; AFR, African; AMR, admixed American; alt base, altered base at the defined position; C, cytosine; CA, controls with asymptomatic pregnancies (at least one pregnancy with noNVP); chrom, chromosome; EAS, East and South Asian combined; EUR, European; G, guanine; GFRAL, glial cell-derived neurotrophic factor receptor alpha-like; HG, hyperemesis gravidarum; HT, HG-TPN (severe hyperemesis gravidarum requiring total parental nutrition); NVP, nausea and vomiting in pregnancy; pos, position; ref base, base at the defined position in the reference genome; sift, sorting intolerant from tolerant; T, thymine.

a HT (severe HG requiring total parenteral nutrition), CA (at least one pregnancy with noNVP).

### Rare and novel *GFRAL* variants selectively detected in controls

Our study was not powered for a meaningful quantitative enrichment analysis in HG cases versus controls. But individuals totally unaffected by NVP are also uncommon. There may be evolutionary disadvantages at both ends of the clinical spectrum. Therefore, assuming that *GFRAL* variants may also be associated with decreased NVP risk, we analyzed variants occurring uniquely in controls. 11 variants (absent in HG cases) were identified (Table 1), including six missense (one novel), four intronic (two novel), and one frameshift. Although none affect known GDF-15 interaction sites,^21^ three missense variants (rs200268366, rs962296758, and rs199980249) were located at the ligand-binding domain intersection, possibly impacting GFRAL function. The frameshift variant rs527905870 was identified in two controls who both reported a pregnancy without NVP, suggesting that defective GFRAL may protect against NVP. Still, given the small numbers and absence of genome-wide benchmarking, these case-exclusive and control-exclusive variants should be interpreted as exploratory signals rather than evidence of enrichment or causality.

### *GFRAL* HG-risk variant and prolonged symptom duration

With NVP, symptoms generally resolve in the second trimester, but can last longer in pregnancies affected by HG. To determine whether a common non-coding HG-risk variant upstream of *GFRAL* (rs7761177-T) is associated with symptom duration, we genotyped the locus in 38 HG cases self-reporting symptom resolution by 27 weeks and in 87 patients whose symptoms lasted longer than 27 weeks’ gestation. The risk allele was associated with 1.7-fold increased risk of prolonged HG (OR = 1.74, CI = 1.01–3.01; *p* = 0.047) (Table S2). Given the modest sample size and marginal *p* value, this association should be viewed as preliminary and will require replication in larger cohorts. In 40 subjects we further sequenced the frequently co-inherited variant rs10948901-T as a proxy for rs7761177. The reason a proxy was used is that rs10948901 was more recently identified in a large multi-ancestry genetic study of HG to be the strongest genetic risk variant in *GFRAL*. So we switched to the variant since the recent evidence suggests it is more likely to be causal and generalizable across ancestries than the originally identified and closely linked variant rs7761177.^18^ However, there was no detectable correlation between the risk variant(s) and sGFRAL levels.

### sGFRAL detection rates by trimester in HG and controls

Recently the GDF-15-GFRAL system has been shown to be susceptible to desensitization,^10^ which could help explain why nausea and vomiting are uncommon in the third trimester (T3) even though GDF-15 levels peak in T3. The mechanism for desensitization is unknown. Currently, GFRAL surface expression or signaling in the human brainstem during pregnancy cannot be measured. While GFRAL cleavage may be involved, sGFRAL resulting from shedding^10^ had never been analyzed in pregnant women. We thus developed an in-house ELISA to measure circulating GFRAL in serum and plasma from women with and without HG. The 3P10 antibody^22^ used for capture binds GFRAL at the RET binding interface, centered around GFRAL residue Arg294. While we cannot exclude further sGFRAL species beyond the 333 amino acid MMP14 cleavage fragment, any sGFRAL fragment captured by 3P10 in an ELISA will leave behind a truncated GFRAL incapable of signaling via RET. The resulting loss-of-function phenotype regarding brainstem GFRAL/RET signaling is expected to prevent GDF-15/GFRAL-induced HG or NVP induction. sGFRAL detection rates were 37% in non-pregnant, 47% in first and second trimester, and 41% in third trimester pregnant women (Figure 1; Table S1, no statistically significant differences). In non-pregnant females, sGFRAL was detected in 46/125 serum samples (37%), which was not significantly different from the 133 pregnant individuals analyzed in T1 (47%) (OR = 0.65, 95% CI = 0.39–1.06, *p* = 0.09) (Figure 1).

**Figure 1:**
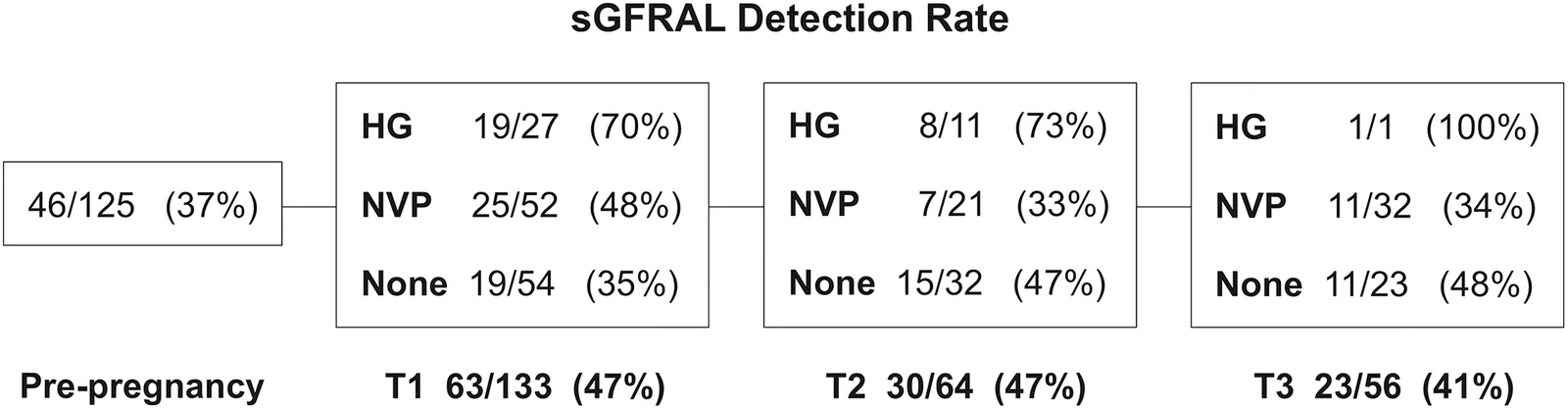
sGFRAL can be present in sera from pregnant women Detection rate of circulating cleaved GFRAL (sGFRAL) in pre-pregnancy and in first (T1), second (T2), and third trimester (T3) in patients with hyperemesis gravidarum (HG), normal nausea and vomiting of pregnancy (NVP), and no nausea and vomiting (noNVP) using a customized in-house sGFRAL ELISA.

In the first trimester, sGFRAL detection rates were, however, significantly higher in HG cases (19/27; 70%) than in non-HG women (44/106; 42%) (OR = 3.35, 95% CI = 1.34–8.33, *p* = 0.009) or in noNVP pregnancies (19/54, 35%) (OR = 4.38, 95% CI = 1.61–11.86, *p* = 0.004). We thus wondered whether receptor shedding might reflect increased GFRAL activity. Despite the small sample size (*n* = 8), the MSC cohort revealed an inverse correlation between sGFRAL levels and weight loss in early pregnancy (R^2^ = 0.44, |r| ≈ − 0.67, 95% CI: −0.93 to 0.09, *p* = 0.005), which is consistent with a protective role of receptor shedding (Figure 2). At later stages of pregnancy, the association became weaker. To really analyze potential correlations between sGFRAL and weight loss in HG patients over the course of pregnancy, a larger cohort would be needed. Weight loss was not observed in non-HG cohorts.

**Figure 2:**
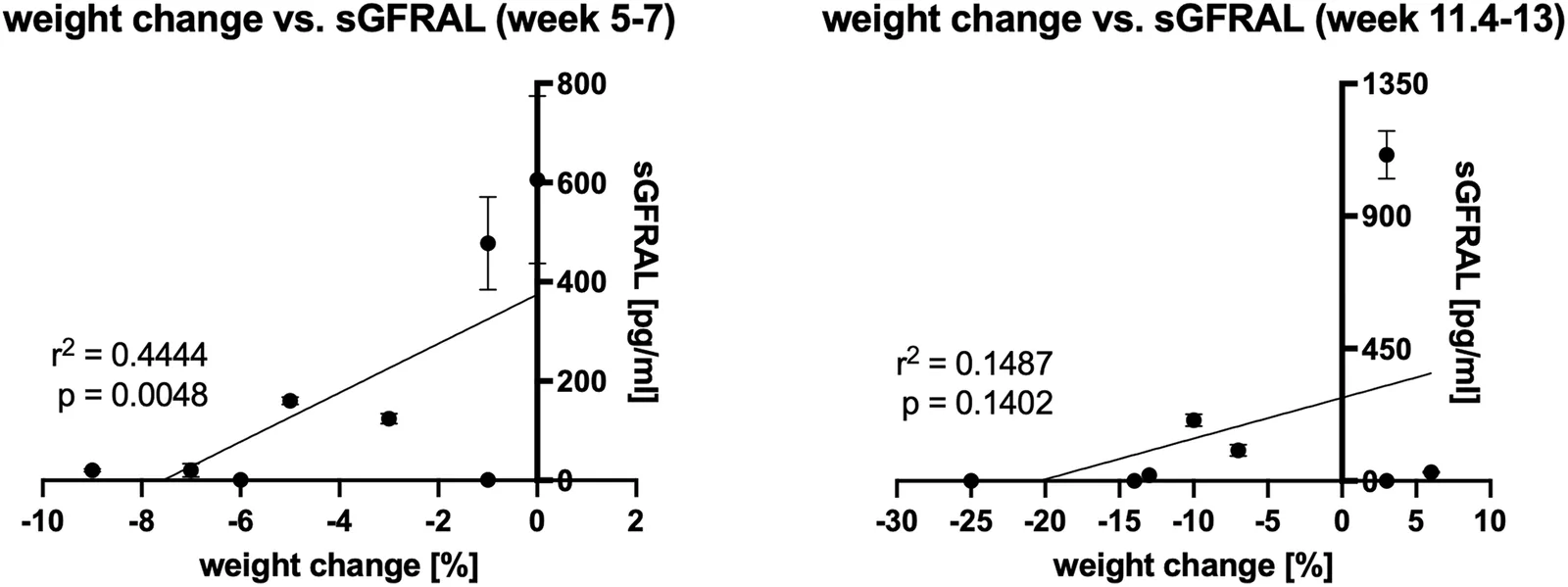
Correlation between weight loss and circulating GFRAL (sGFRAL) during early pregnancy in women receiving intravenous fluid treatment due to hyperemesis gravidarum An in-house developed sGFRAL ELISA was used to measure serum samples (*n* = 8 per time point, measured in duplicate). Solid lines represent linear regression fits. Horizontal bars indicate mean ± SD from technical duplicates. Where error bars are invisible, they are overlaid by the symbols. Coefficients of determination (R^2^) and *p* values are indicated. ELISA, enzyme-linked-immunosorbent assay; GFRAL, glial cell-derived neurotrophic factor receptor alpha-like; SD, standard deviation.

### Circulating sGFRAL levels in HG, NVP, and noNVP cases at 12 and 24 weeks’ gestation

We compared mean serum sGFRAL levels in 11 hospitalized first-trimester HG cases at two time points, 12 weeks’ gestation (*N* = 10, one missing sample) and 24 weeks’ gestation (*N* = 11) to those in women reporting NVP in the first trimester (*N* = 21 at both time points) and women with noNVP (*N* = 31 at 12 weeks’ gestation, *N* = 32 at 24 weeks’ gestation). sGFRAL levels ranged from undetectable to 1563 pg/mL (mean = 77 pg/mL) during pregnancy and from undetectable to 4179 pg/mL (mean = 163 pg/mL) in non-pregnant participants of reproductive age. A two-part model was applied to account for the high proportion of values below the limit of detection. In the first part (logistic regression for detectability), time point (24 vs. 12 weeks) had no significant effect (OR 0.91, 95% CI 0.38–2.14, *p* = 0.823). Across all measurements (12 and 24 weeks combined), women with HG had significantly higher odds of detectable sGFRAL than noNVP (OR 3.03, 95% CI 1.11–8.72, *p* = 0.034), whereas NVP did not differ from noNVP (OR 1.87, 95% CI 0.41–8.47, *p* = 0.598). Time-specific analyses at 12 and 24 weeks showed similar trends for higher detection rates in HG, but these did not reach statistical significance (Figure 3B).

**Figure 3:**
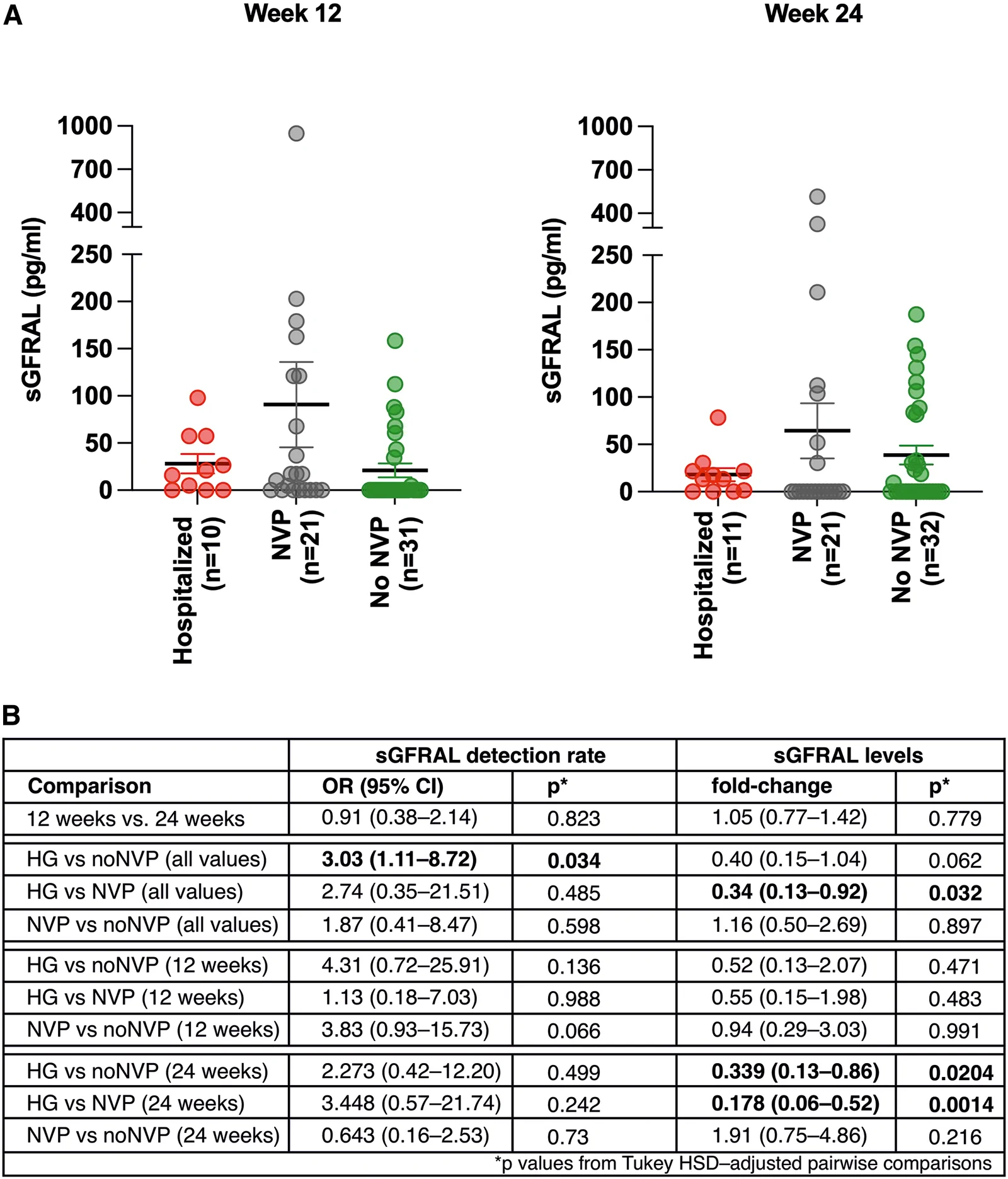
Circulating soluble GFRAL (sGFRAL) in the CGATE cohort at 12 and 24 weeks’ gestation and summary of two-part analyses (A) Serum sGFRAL concentrations (pg/mL) in women with hyperemesis gravidarum (HG), nausea and vomiting of pregnancy (NVP), and no nausea and vomiting (noNVP) at 12 and 24 weeks’ gestation, measured by in-house ELISA and displayed as scatter dot plots indicating the mean with SEM. Values below the lower limit of detection (LOD = 10 pg/mL) are plotted at 1 pg/mL for visualization. (B) Results of a two-part modeling approach accounting for the high proportion of values below the LOD. Columns 2 and 3 (“sGFRAL detection rate”) show odds ratios (ORs) and *p* values from logistic regression models for detectable sGFRAL (≥LOD), including the overall time effect (24 vs. 12 weeks), group comparisons averaged over both time points (“all values”), and group comparisons at 12 and 24 weeks separately. Columns 4 and 5 (“sGFRAL levels”) show fold-changes in sGFRAL concentrations from linear mixed-effects models among samples with detectable sGFRAL, again reported for the overall time effect, for group comparisons across all values, and for group comparisons at each time point. Across all values, HG showed higher odds of detectable sGFRAL than noNVP, whereas among detectable samples, sGFRAL concentrations were lower in HG than in NVP and noNVP, particularly at 24 weeks (see table for ORs, fold-changes, 95% CIs, and *p* values).

The second (mixed linear) part of the model included only samples with detectable sGFRAL concentrations. At 12 weeks, sGFRAL levels showed a weak non-significant trend toward lower values in HG compared with NVP and noNVP (e.g., fold change vs. noNVP 0.52, *p* = 0.471). By 24 weeks, detectable sGFRAL concentrations were significantly lower in HG than in both NVP (fold change 0.178, *p* = 0.0014) and noNVP (fold change 0.339, *p* = 0.0204), with no significant difference between NVP and noNVP. Pooled analyses across all values were directionally consistent with these time-specific contrasts. Results are summarized in Figure 3.

### Circulating sGFRAL is transiently detectable in early noNVP pregnancies

Early cleavage of GFRAL without compensatory GFRAL synthesis provides a plausible model that could help explain why women with “noNVP” pregnancies do not experience GDF-15-GFRAL-mediated symptoms. However, collections of sera from the first weeks of naturally conceived pregnancies are limited. We thus analyzed ten available samples from an IVF pregnancy cohort with normal pregnancy outcomes (Figure 4A). None were diagnosed with HG, but two took anti-emetics (depicted in red), with one of the two taking sick leave due to nausea. sGFRAL was detectable in 8/10 of these samples (mean: 218.7 pg/mL, median: 149.42 pg/mL, Figure 4B). Strikingly, at a second sampling (between day 62 and 76), none of the eight analyzed women (including the one taking anti-emetics without sick leave) showed detectable sGFRAL levels any more. Only the patient on sick leave, who had her second sampling on day 103, still showed high sGFRAL. Excluding the two patients taking anti-emetics, the difference in sGFRAL levels between first and second sampling was statistically significant (*p* = 0.032). Early near-complete cleavage of membrane-bound GFRAL would be compatible with efficient desensitization of the GDF-15-GFRAL pathway, for example through GFRAL shedding and/or reduced new GFRAL synthesis, and may thus explain desensitization to GDF-15-induced NVP or even HG in at least a subset of pregnancies. Still, this inference requires confirmation in larger, prospectively sampled cohorts.

**Figure 4:**
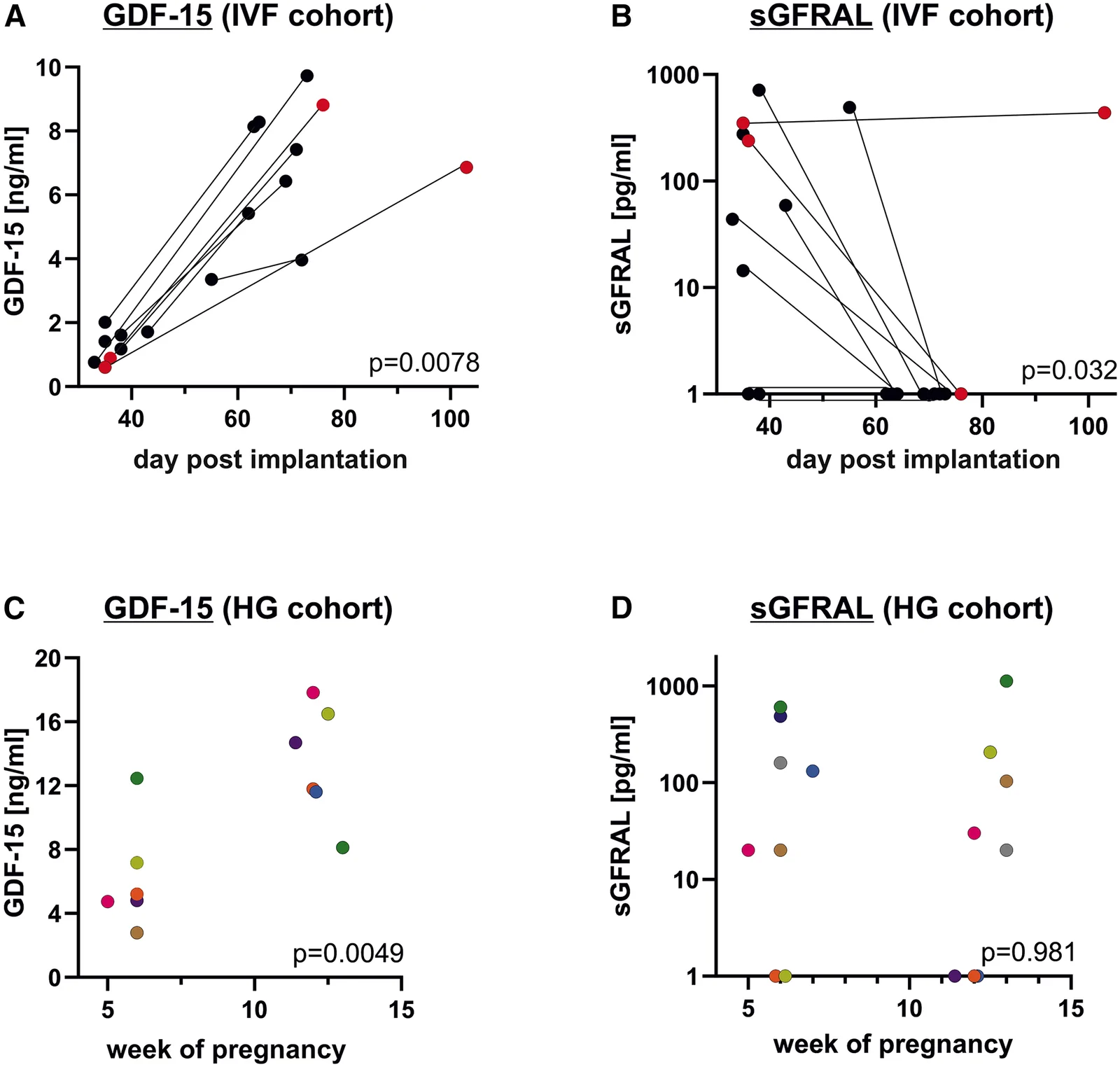
GDF-15 and soluble (s) GFRAL levels in “healthy” pregnant women and women with hyperemesis gravidarum (A and B) GDF-15 and sGFRAL levels were analyzed in ten samples from an *in vitro* fertilization (IVF) pregnancy cohort (without HG diagnosis) collected at two time points: first sampling between day 35 and 43 post-implantation and second sampling between day 62 and 76, or on day 103 for the patient who went on sick leave due to nausea. 2/10 women (depicted in red) took anti-emetics. Statistical analysis was performed using the Wilcoxon signed-rank test (excluding the two women taking anti-emetics). (C and D) GDF-15 and sGFRAL levels were analyzed in serum samples from pregnant women with HG (collected at six weeks’ and 12 weeks’ gestation). Statistical analysis was performed using paired Student’s *t* test. GDF-15, growth/differentiation factor 15; GFRAL, glial cell-derived neurotrophic factor receptor alpha-like.

### Circulating sGFRAL levels are sustained in HG patients

Having found that GFRAL shedding tends to occur early in unaffected pregnancies, with sGFRAL becoming undetectable by around week eight, we analyzed serum from patients requiring intravenous fluid treatment for HG-related dehydration. MSC provided eight serum samples collected at six weeks’ and eight at 12 weeks’ gestation. Samples were matched for participant race and pre-pregnancy weight. In stark contrast to the IVF cohort, this comparison revealed no drop in sGFRAL (mean at 1^st^ sampling: 177.7 pg/mL, median: 75.74 pg/mL, mean at 2^nd^ sampling: 211.4 pg/mL, median at 2^nd^ sampling: 30.14 pg/mL, *p* = 0.981) (Figures 4C and 4D). This may reflect persistent GFRAL synthesis in HG and/or more effective suppression of new synthesis in unaffected pregnancies.

## Discussion

### Main findings

This study explores the role of GFRAL and sGFRAL in HG. First, we report rare *GFRAL* variants that may modulate HG susceptibility. All variants except p.Ile353Val and p.Met370Thr are in the extracellular domain. Glycosylation sites, disulfide bridges, the MT1-MMP cleavage site and the GDF-15 contact residues Ile196, Pro197, Gln200, Ser201, and Ala204 are not affected.^21^ However, p.Gln199Arg and p.Glu203Ala are immediately adjacent to the GDF-15 contact residues Gln200 and Ala204, respectively. The variant rs138536707 identified in African American patients is of particular interest because people of African ancestry are at increased risk for HG.^1^ Further study is needed to determine whether this rare variant located in the 5′UTR has any affect on transcription, stability, or translation. The “control-only variants” p.Pro218Ala, p.Ser222Asn, and p.Arg225His are at the intersection of the GDF-15 interaction (aa149-228) and the GDNF/GAS1 domain (220–316), which are responsible for ligand binding and signal transduction, potentially affecting GFRAL functionality. The p.Ile339AsnfsTer36 (rs527905870) variant identified in 2 women who had pregnancies with noNVP, was recently shown to be a loss of function variant associated with increased alcohol consumption, lending further support that blocking this pathway may effectively reduce an aversive response and improve appetite in patients with HG.^23^ The p.Met370Thr variant identified in the patient requiring total parenteral nutrition lies close to the membrane-proximal region of GFRAL and could affect the interaction of GFRAL with RET. Replacing the bulkier hydrophobic methionine with threonine introduces a polar side chain that could form additional hydrogen bonds and allow slightly tighter local packing between GFRAL and RET. While still hypothetical, this would be consistent with a subtle stabilization of the GFRAL-RET interaction, which could slow dissociation of the signaling complex and maintain RET engagement even after GFRAL cleavage at R333-K, in line with the observed severe HG. In-depth functional and biochemical characterization of this and other identified GFRAL mutants represents a promising direction to elucidate molecular mechanisms underlying HG. It is important to note that the observed case-exclusive and control-exclusive variants cannot be interpreted as evidence of enrichment or causality. These rare variants are therefore best interpreted as hypothesis-generating signals, rather than as definitive disease alleles. While their current value lies in suggesting candidate mechanisms and *loci* for follow up, further studies in larger and more diverse populations, coupled with mechanistic analyses, will be required to determine the functional significance of these variants. Still, our data already indicate that variations in GFRAL are too infrequent to account for the majority of HG or no-NVP pregnancies. We further determined that a common *GFRAL* HG-risk variant is significantly linked to prolonged symptoms suggesting a possible role in disrupting desensitization. However, it did not correlate with sGFRAL levels, so the mechanism remains unknown. These results highlight the potential involvement of *GFRAL* variants in HG pathogenesis, but more work is needed.

To address this gap, we complemented the genetic data with disease-contextual analysis of circulating sGFRAL in humans, providing preliminary evidence for its potential clinical relevance in pregnancy and in HG. Specifically, sGFRAL was detected at roughly 2-fold higher rates in HG cases compared to women with noNVP in the late first trimester when nausea and vomiting symptoms peak (detection rates: 70% vs. 35%, OR: 4.38, 95% CI: 1.61–11.86). Based on the CGATE cohort from mid-gestation, a two-part regression framework found that, across 12 and 24 weeks combined, women with HG had higher odds of detectable sGFRAL than noNVP, whereas NVP did not differ from noNVP. Among samples with detectable sGFRAL, mixed-effects models showed that sGFRAL concentrations were significantly lower in HG than in NVP and lower than, or trending lower than, in noNVP, particularly at 24 weeks. Taken together, these patterns indicate that HG differs from NVP and noNVP both in the likelihood and in the magnitude of circulating sGFRAL.

Longitudinal analysis from earlier time points extends these observations. IVF-conceived non-HG pregnancies showed sGFRAL in 8/10 blood samples obtained in week five to six (mean: 218.7 pg/mL, median: 149.42 pg/mL), whereas the only sample still showing detectable sGFRAL levels at week eight or later was from the only patient who went on sick leave due to nausea. In comparison, an HG cohort showed very similar (just slightly lower) sGFRAL levels around week six (mean: 177.72 pg/mL, median: 75.74 pg/mL, *p* = 0.72) but no drop in sGFRAL levels in week 12 serum samples. Sustained sGFRAL levels in blood may thus reflect less effective cleavage at early time points, and/or increased GFRAL expression and/or activity in the brainstem of HG patients.

Alternatively, more frequent sGFRAL detection in HG cases might be the result of enhanced, rather than protracted GFRAL cleavage. Membrane-bound MT1-MMP, the only enzyme known to cleave GFRAL,^10^ is induced by inflammation, which is also associated with HG.^24^ A protective function of GFRAL cleavage is supported by the inverse correlation between sGFRAL and weight loss in the HG cohort. Altogether, our data suggest several yet-to-be confirmed, but biologically plausible determinants of GDF 15-GFRAL-RET signaling activity: (1) variations in *GFRAL* may cause pathway inactivation, hyperactivity, and prolonged symptoms. (2) Shedding of membrane-bound GFRAL occurs in human pregnancy, likely leaving behind an inactive receptor fragment on brainstem neurons. (3) The heterogeneous NVP cohort comprises many individuals with undetectable sGFRAL, but also those with the five highest sGFRAL levels. High shedding activity may protect these latter patients from HG, despite high GFRAL availability. HG patients, in contrast, show a distinct pattern with higher detectability but lower detectable concentrations of sGFRAL, indicating that GFRAL regulation in HG differs from NVP and noNVP, although we cannot determine from these data whether this reflects changes in shedding, synthesis, or both. (4) While replacement of cleaved GFRAL cannot be measured, the association between sustained sGFRAL levels and HG suggests that new GFRAL synthesis may be lowest in noNVP pregnancies. (5) Sustained presence of sGFRAL in blood may be explored as a novel biomarker for HG. *In silico* analysis (Figure S1) further shows an Sp1-rich GFRAL promoter with progesterone response elements and a non-canonical estrogen responsive element.^25^^,^^26^ This predicted promoter would be highly susceptible to progesterone receptor-mediated transcriptional repression. Hormonal suppression of *de novo* GFRAL synthesis could thus explain the association between genetic variants in the progesterone receptor and HG.^18^ While transcriptional repression may be somewhat leaky, as evidenced by occasional detection of sGFRAL in sera from later-stage normal pregnancies, GFRAL cleavage can then still prevent major discomfort. If validated, analysis of sGFRAL could provide a novel biomarker for HG diagnosis and screening, as well as for related conditions involving the GDF-15-GFRAL pathway, such as cancer cachexia and chemotherapy-induced nausea and vomiting.

### Interpretation

Our results provide insight into desensitization to GDF-15-GFRAL signaling. HG results, at least in part, from hypersensitivity to pregnancy-induced GDF-15. Genetic predisposition for lower baseline levels prior to conception is a risk factor, whereas preconceptional chronic tobacco smoking, pre-pregnancy daily metformin use or β-thalassemia—which are associated with elevated GDF-15^27^^,^^28^^,^^29^ —are linked to reduced HG risk.^17^^,^^30^^,^^31^ By providing initial evidence for sGFRAL in sera/plasma from pregnant women, we add a second, receptor level dimension to this model. Early and near-complete GFRAL shedding in uncomplicated pregnancies is compatible with efficient GFRAL shedding and/or downregulation of new GFRAL synthesis, whereas the persistence of detectable sGFRAL in HG and the mid-gestation pattern (higher detectability but lower detectable concentrations in HG than in NVP and noNVP) suggest altered dynamics of GFRAL shedding and/or synthesis in HG. Mechanistically, any 3P10-reactive ectodomain detected as sGFRAL implies the presence of a truncated membrane receptor that is unlikely to signal via RET. It is therefore plausible that sGFRAL reflects desensitization of the GDF-15-GFRAL pathway, with early and efficient shedding, incomplete recovery of intact GFRAL, or both, potentially contributing to reduced susceptibility to HG in some pregnancies. Conversely, less efficient desensitization could predispose to HG or prolong symptoms. However, the present data cannot distinguish between altered shedding and altered *de novo* synthesis, and we cannot directly measure intact GFRAL on brainstem neurons in pregnant women. All mechanistic interpretations regarding “cleavage efficiency” or “recovery” should therefore be regarded as hypothesis generating. The conceptual framework developed from our observations remains to be tested in future mechanistic and longitudinal studies.

Despite these limitations, our results already have important implications for HG therapeutics. A clinical trial investigating GFRAL inhibition with the monoclonal antibody NGM120 is currently underway in the UK (ISRCTN76681798) and Australia (ACTRN12624001421527). Future research should explore whether *GFRAL* variants or circulating sGFRAL can serve as predictive biomarkers of treatment response. Furthermore, engineered sGFRAL mimetics acting as GDF-15 traps could become novel therapeutics not only for HG but also for other GDF-15-driven conditions, including cachexia and chemotherapy-induced nausea and vomiting.

These exploratory findings suggest that GFRAL signaling in normal pregnancy and HG can be modulated by shedding. Hypothesis-generating findings include new genetic variants and potential biomarkers for risk stratification and treatment response, and highlight the GDF-15-GFRAL pathway as a plausible target for future therapeutic approaches. Large-scale and mechanistic studies will be essential to confirm these insights and explore therapeutic targeting of the GDF-15-GFRAL pathway.

### Limitations of the study

This study includes genetic and serological analyses across multiple well-characterized, but partly small cohorts. Race and ethnicity were not recorded in the European cohorts (CGATE, IVF, PMOS, non-pregnant controls). Ancestry-stratified analyses were therefore not possible for these cohorts. The CGATE and non-pregnant control cohorts approximate unselected populations from defined catchment regions (Franconia, Germany, and Umeå, Sweden), whereas the IVF and PMOS cohorts were clinically selected; generalizability of biomarker findings across ancestral backgrounds requires confirmation in cohorts with recorded ethnicity or genetic ancestry data. Signal-seeking analyses detect novel *GFRAL* variants including one specifically increased in African American women with HG and find an association of a common *GFRAL* variant with prolonged HG. These findings suggest, but do not prove a possible mechanistic role for GFRAL cleavage in pregnancy and HG, and imply that sGFRAL dynamics may hold potential as a biomarker for HG. The 3P10 capture antibody used for ELISA capture binds GFRAL at the RET-interaction domain around R294,^32^ while the canonical MMP14 cleavage occurs at (R333-K).^10^ The MMP14-cleaved extracellular N-terminal fragment thus comprises both the RET-binding region and the 3P10 epitope. The existence of additional sGFRAL species cannot be excluded. However, any 3P10-reactive sGFRAL fragment will leave behind a dysfunctional membrane-bound receptor fragment incapable of RET signaling. Accordingly, sGFRAL was used as proxy for proteolytic receptor inactivation. Currently, there is no method available for directly measuring intact GFRAL at the cell surface of brainstem neurons in pregnant women.

Key limitations are low variant frequencies, limited third-trimester HG samples, and the inability to fully assess early gestational adaptation. Replication in larger cohorts and functional validation are needed before any evidence of pathological significance can be concluded. Analysis of additional cohorts across early to late gestation may further support adaptation of the GDF-15-GFRAL pathway by cleavage. At present, our genetic and serological findings should therefore be regarded as exploratory and mechanistic hypotheses that motivate targeted functional studies, rather than as definitive demonstrations of causality. Still, even a larger study would not be able to directly monitor the level of active, non-cleaved GFRAL at the brainstem of pregnant women. HG is difficult to model in small animals, as mice and other rodents lack the vomiting reflex.^33^ Therefore, some gaps are challenging to address experimentally. Nevertheless, we hope that this pilot study will spur further research on GFRAL regulation in pregnancy.

## Resource availability

### Lead contact

Requests for further information and resources should be directed to and will be fulfilled by the lead contact, Jörg Wischhusen (wischhusen_j@ukw.de).

### Materials availability

The antibodies and sGFRAL standard used in the ELISA are commercially available. Requests for clinical samples must be addressed to the respective medical centers. Availability of surplus materials and permission to share them cannot be guaranteed. Analyses of human samples may require ethical approval from the respective committee. The raw data supporting the findings of this study have been uploaded to Mendeley. The expression plasmid for sGFRAL-Fc is available from the lead contact with a completed materials transfer agreement.

### Data and code availability

• Raw [.xml] data derived from human samples have been deposited at Mendeley (Wischhusen, Jörg (2026), “GFRAL genetic variations and circulating GFRAL in hyperemesis gravidarum. Haack et al.”) and are publicly available [https://doi.org/10.17632/czzbkky3cn.1].
• This paper does not report original code.
• Any additional information required to reanalyze the data reported in this paper is available from the lead contact upon request.

## Acknowledgments

This work was supported, in part, by an external patient and scientific peer-reviewed grant (MSF, KWM) from the Bikkja Trust, who did not play a role in research or manuscript preparation. It was further supported by external scientific peer-review grants from the 10.13039/501100005416Research Council of Norway (no. 213497), 10.13039/501100002960Västerbotten County Council (no. 310426013), 10.13039/501100007067Kempestiftelserna (JCSMK24-0030), the 10.13039/501100003748Swedish Society for Medical Research (no. P16-0062), Umeå University Foundations (FS.2.1.6-1577-17), EU FP7-People-Cofund (INCA 600398), the 10.13039/501100004359Swedish Research Council (2015-00495; 2016-01439; 2020-01801), the 10.13039/501100007687Swedish Society of Medicine (SLS-573171, SLS-597211, and SLS-789101), and the 10.13039/501100003792Swedish Brain Foundation (2020-0255) who neither did play a role in the research or manuscript preparation. Further support was provided by a German/European grant #BMBF01KT2310 TRANSCAN-3 (ERA-NET): Sustained collaboration of national and regional programmes in cancer research—JTC 2021) iParaCyts to J.W., which was externally reviewed. This work was also funded by the 10.13039/501100001659Deutsche Forschungsgemeinschaft (DFG, German Research Foundation): KFO 296, 255154572 and SFB 1713; 534829736 to M.A.F., which was externally reviewed. The funding bodies had no impact on the content of research. We also acknowledge support from Dr. Lars Schönemann (Recombinant Protein Expression Facility at the University of Wuerzburg) for in-house production of sGFRAL-Fc. The funding bodies had no role in study design, data collection, data analysis, data interpretation, or writing of this manuscript.

## Author contributions

Conceptualization: J.W., M.S.F., M.B., M.N., and M.-T.V.; generation of reagents and assay development: B.H., F.H., and J.W.; patient recruitment, provision of patient samples with medical records, and data curation: S.D., K.W.M., P.A.F., A.H., E.V., M.B., L.S., and E.E.; experimental investigation: B.H., Z.H.N., and B.F.; formal analysis: M.-T.V., J.W., and M.S.F.; writing (draft): M.S.F., M.-T.V., and J.W.; writing (review and editing): B.H., Z.H.N., S.D., K.W.M., B.F., F.H., P.A.F., A.H., E.V., M.A.F., M.B., L.S., E.E., M.N., M.-T.V., J.W., and M.S.F.; M.S.F., J.W., B.H., and M.-T.V. accessed and verified the underlying data. All authors had full access to all the data in the study and accept responsibility to submit for publication.

## Declaration of interests

B.H., Z.H.N., S.D., K.W.M., B.F., F.H., P.A.F., E.V., M.B., L.S., E.E., M.N., and M.-T.V. have nothing to disclose. M.A.F. has received honoraria for consultations from Alexion, Kyverna, Lundbeck, Merck KGaA, neuraxpharm, Novartis, Roche, and Sudo Biosciences. He is a member of the advisory board of Wings for Life. He holds stocks from Bayer and NovoNordisk. J.W. is an inventor on patents related to GDF-15 as a biomarker and therapeutic target in cancer, and a co-founder and shareholder of the biotech company CatalYm GmbH involved in translating GDF-15-based cancer therapies and diagnostics into the clinics. A.H. and M.S.F. are paid consultants for NGM Bio. M.S.F. is also a shareholder of NGM Bio.

## Declaration of generative AI and AI-assisted technologies in the manuscript preparation process

During the preparation of this work the authors used Biomed Advisor, a dedicated Scientific AI tool, in order to support the *in silico* analysis of the GFRAL promoter region, and to generate a graphical abstract. After using this tool, the authors reviewed and edited the content as needed and take full responsibility for the content of the published article.

## STAR★Methods

### Key resources table

**Table:** 

REAGENT or RESOURCE	SOURCE	IDENTIFIER
**Antibodies**		

human monoclonal anti-GDF-15 (clone B1-23)	Lonza	N/A; patent EP3122775B1; 2 μg/mL
mouse monoclonal anti-GFRAL (clone 3P10) described in Suriben et al.^32^	Evitria	N/A; 2 μg/mL
sheep polyclonal anti-GFR alpha-like Isoform A	R&D Systems	Cat#BAF5728; RRID: AB_2044677; 50 ng/mL

**Biological samples**		

DNA, serum and plasma samples from pregnant women with either *hyperemesis gravidarum* or nausea and vomiting of pregnancy or no nausea or vomiting	Clinical centers contributing to this study as indicated in this manuscript	Samples were not commercially available (no commercial identifier)

**Chemicals, peptides, and recombinant proteins**		

HRP Streptavidin	BioLegend	Cat#405210; 1:1000
Bovine Serum Albumin (BSA) Fraction V, NZ-Origin, biotin-free	Carl Roth	Cat#0163.2; CAS: 9048-46-8
NheI-HF(R)	New England Biolabs	Cat#R3131S
NotI-HF(R)	New England Biolabs	Cat#R3189S
Sulfuric Acid, 98%	Carl Roth	Cat#X945.1; CAS: 7664-93-9
Tween®20	Carl Roth	Cat#9127.2; CAS: 9005-64-5
Recombinant Mouse GFR alpha-like Fc Chimera Protein	R&D Systems	Cat#9844-GR
cOmplete™, EDTA-free Protease Inhibitor Cocktail	Merck	Cat#4693132001
Sodium dihydrogen phosphate dihydrate	Carl Roth	Cat#T879.2; CAS: 13472-35-0
Di-sodium hydrogen phosphate dihydrate	Carl Roth	Cat#4984.1; CAS: 10028-24-7
Sodium chloride	Carl Roth	Cat#P029.2; CAS: 7647-14-5
Glycine	Sigma-Aldrich	Cat#G7126; CAS: 56-40-6
Tris	Carl Roth	Cat#A411.2; CAS: 77-86-1
Phosphate-buffered saline (PBS)	Sigma-Aldrich	Cat#D8537
Expi293™ Expression Medium	ThermoFisher Scientific	Cat#1435101

**Critical commercial assays**		

Human GDF-15 DuoSet ELISA	R&D Systems	Cat#DY957
Total GDF-15 enzyme linked immunosorbent assay (ELISA) kit	AnshLabs	Cat#AL-1014-r
TMB Substrate Set	BioLegend	Cat#421101
Expi293™ Expression System Kit	ThermoFisher Scientific	Cat#A14635
TaqMan Assay	ThermoFisher Scientific	Assay ID C_189464873_10

**Deposited data**		

Raw and analyzed data	This paper; Mendeley Data	https://doi.org/10.17632/czzbkky3cn.1

**Experimental models: Cell lines**		

ExpiF Human Embryonic Kidney Cells	ThermoFisher Scientific	Cat#14527

**Oligonucleotides**		

Primers for rs138536707 Forward: TAGGAAGCAAACACAGAGGC, Reverse: GCCCCTGTTAAGCTTAAGTGT	This paper	N/A
Primers for rs10948901 Forward: TTGCCAAGGTGTGAGGTCTA, Reverse: ACTTATTGGGCTTTGCCAGC	This paper	N/A
TaqMan manufacturer-provided VIC/FAM target sequence for genotyping rs7761177:GAAGGTTAACACTGGTACAGAAATA [T/C]TTCATAAAAGTTATGAAAACCAGAT	Thermo Fisher Scientific	Assay ID C_189464873_10

**Recombinant DNA**		

pEF1a-GFRAL(Ser19–Glu351)–IEGRMD–human IgG1 Fc (codon-optimized)	Twist Bioscience	Table S4
pEF-1 alpha expression plasmid	Twist Bioscience	N/A

**Software and algorithms**		

GraphPad Prism 10.3.1	GraphPad Software, Inc. (Boston, USA)	https://www.graphpad.com; RRID: SCR_000306
Magellan^TM^ Software 7.5	Tecan (Männedorf, CH)	https://lifesciences.tecan.com/software-magellan?p=tab--1
4Peaks	Nucleobytes	http://nucleobytes.com/index.php/4peaks;RRID: SCR_000015
UNICORN	Cytiva Software	URL https://www.cytivalifesciences.com/en/us/products/items/unicorn-7-p-05649?psmenu=0
MedCalc Software Ltd. Odds Ratio Calculator	MedCalc Software Ltd.	https://www.medcalc.org/en/calc/odds_ratio.php (Version 23.4.2; accessed Dec 19, 2025)
CorelDRAW 25	Corel Corporation	https://www.coreldraw.com/en/; RRID: SCR_014235

**Other**		

Tecan Sunrise Plate Reader	Tecan, Männedorf, CH	N/A
Nunc MaxiSorp 96-Well ELISA microplates	ThermoFisher	Cat#442404
Whole-exome sequencing	Fejzo et al.^34^	https://pubmed.ncbi.nlm.nih.gov/35218128/
Nalgene™ Single-Use PETG Erlenmeyer Flasks with Plain Bottom: Sterile	ThermoFisher Scientific	Cat#4115-0125
Filtropur S 0.2	Sarstedt AG & Co. KG	Cat#831.1826.001
ÄKTA Purifier 10 FPLC system	Amersham	Cat#28-4062-64
HiTrap™ Protein G HP antibody purification columns	Cytiva	Cat#29048581
Amicon® Ultra Centrifugal Filter, 50 kDa MWCO	Merck	Cat#UFC805008

### Experimental model and study participant details

#### Cell lines

The human embryonic kidney cell line (ExpiF) was purchased from ThermoFisher Scientific and maintained in suspension culture in Expi293 Expression Medium (ThermoFisher Scientific). For cell line authentication, ThermoFisher utilizes its own industry-standard fragment analysis technology (akin to standard Short Tandem Repeat (STR) Profiling) to map 16 to 24 highly variable microsatellite loci plus the Amelogenin gender marker to generate a unique DNA profile. Cells were grown in flasks on a shaker platform at 37°C in a humidified incubator with 8% CO_2_. The cell line was tested negative for mycoplasma contamination.

#### Human study participants

All study subjects were females of reproductive age. Cohort size, age range and mean age are indicated for each individual cohort below. Data on race and ethnicity are provided for the cohorts from the US. These data were not available for study participants from Europe. Participants were assigned to the respective group (noNVP, NVP, HG) based on their medical records for HG and self-reported symptoms for noNVP and NVP. All study participants provided informed consent.

#### Clinical cohorts

##### Cohorts used for whole-exome sequencing to identify GFRAL variants unique to HG and controls

HER Cohort: This is a post-hoc analysis of a published WES study that identified an exome-wide significant association in *GDF15* but not *GFRAL*.^18^ Rare variants may contribute to disease burden but not reach exome-wide significance, which was also the case for an additional rare variant in *GDF15* that we reported in the previous study.^34^ The purpose of the current study is to report on rare variants identified in *GFRAL* that occurred only in HG cases and not in unaffected controls and vice versa, providing knowledge from this unique cohort that may be applied to future replication studies. Briefly, the cohort included 926 non-blood-related HG cases recruited through the Hyperemesis Education and Research (HER) Foundation website from 2007 to 2017 (18–39 years old, mean: 34 years, 91% European white, 9% other ethnicities), and 660 non-blood-related controls with at least two pregnancies beyond 20 weeks gestation (28–49 years old, mean: 36 years, 94% European white, 6% other ethnicities). All participants included in the study were predicted to be unrelated using the Regeneron pipeline described previously.^35^ Medical records of intravenous fluid treatment were requested for all cases. The study was approved by the University of Southern California Institutional Review Board (#HS-20-00500).

African American Cohort: *GFRAL* variant rs138536707 (identified in two participants of African Ancestry in the HER cohort) was sequenced in 144 additional African American patients from The HG and Morning Sickness Clinic (MSC) (18–49 years old, mean: 29 years, 100% African American). The study was approved by University of Southern California Institutional Review Board (#HS-21-00312).

##### Clinical cohorts (HG cases and controls) for GDF-15 and sGFRAL analyses

Multiple cohorts provided plasma and serum samples for sGFRAL measurements across pregnancy stages:

The Clinical Gravidity Association Trial and Evaluation (CGATE) Cohort (Germany): A prospective observational study (from 2009 to 2013).^36^ For the present study, 11 women hospitalized with HG (20–38 years old, mean: 29 years), 21 NVP cases (26–33 years old, mean: 30 years), and 32 noNVP controls (22–42 years old, mean: 33 years) were included. Biomaterial was collected at 12 and 24 weeks’ gestation. The study was approved by the Ethics Committee of the medical faculty at Friedrich Alexander University of Erlangen-Nuremberg, Erlangen, Germany (#4176). Data on ethnicity were not recorded.

HG and Morning Sickness Clinic (MSC) Cohort (Birmingham, Alabama): Serum samples from 16 patients in the first trimester (eight at six weeks and eight at 12 weeks' gestation) who received intravenous fluid treatment due to HG were collected (19–34 years old, mean: 28 years; 94% European white, 6% other ethnicities). The study was approved by University of Southern California Institutional Review Board HS-21-00312.

IVF Cohort (Sweden): Ten women undergoing IVF treatment (from 2021 to 2023) with no HG symptoms (27–39 years old, mean: 34 years; ethnicity was not recorded). Six women were healthy, one woman was prescribed Ondansetron, another one Postafen. One woman was diagnosed with PMOS (polyendocrine metabolic ovarian syndrome) and medicated with metformin before and during pregnancy (with a break in early gestation). Another one suffered from overstimulation syndrome. The study was approved by the Swedish Ethical Review Authority (#2013/1324-31/2; #2017/1935-32; #2024-03209-02).

Pregnant PMOS Cohort (Norway, Sweden, Iceland): The pregnant PMOS cohort is a subsample of the PregMet2 study.^37^ 56 pregnant women with PMOS were included: one hospitalised HG case, 32 NVP cases, 23 noNVP cases (22–38 years old, mean: 29 years without the HG patient, 29.5 years including the HG patient; no ethnicity data available). Serum was isolated in the first trimester, prior to treatment, and in the third trimester. The study was approved by the Committee for Medical Research Ethics of Health Region IV, Norway (#145–05); the Regional Committee for Health Research Ethics of Central Norway (#2011/1434), the National Bioethics Committee of Iceland (#VSNb2012100011/03.10), the Regional Ethical Review Board, Stockholm, Sweden (#2012/1200-31/2); and the Regional Ethical Review Board, Umeå, Sweden (#2014-257-31M; #2016-190-32M).

Non-Pregnant Control Cohort (Sweden): The cohort used for the present study was described previously.^38^ Women of 18-45 years (mean: 28 years) were generally healthy except for premenstrual dysphoric disorder (PMDD) in 51.8% of them (based on the local assessment). Serum samples were withdrawn in the mid-follicular phase (day 5-11 of the menstrual cycle) and in the late luteal phase (day −8 to −1). The study was approved by the Regional Ethical Review Board in Umeå, Sweden (#2016-111-31; and #2017-266-32M). Ethnicity data were not recorded.

### Ethics statement

All relevant information regarding the ethical approval committee and ethical approval number is provided in the brief cohort descriptions above.

### Method details

#### Whole-exome sequencing to identify GFRAL variants unique to HG and controls

##### Analysis of GFRAL mutations

Whole exome sequencing of the HER cohort (including recruitment, DNA extraction, whole-exome sequencing by the Regeneron Genetics Center (RGC, Tarrytown, NY, USA), genome informatics, filtering, and ancestry prediction) had been performed in a previous study.^34^ Data were analyzed on the DNAnexus platform (Mountain View, CA, USA) for filtering and identifying rare *GFRAL* variants specific to cases and unaffected controls.

##### Genotyping

*GFRAL* variant rs138536707 (identified in two participants of African Ancestry in the HER cohort) was sequenced in 144 additional African American patients from The HG and Morning Sickness Clinic (MSC). PCR was performed with 2× PCR Master Mix (Catalog #K0171, Thermo Fisher Scientific) final volume 20 µL with 20 ng genomic DNA, and 0.25 μM each of forward primer TAGGAAGCAAACACAGAGGC and reverse primer GCCCCTGTTAAGCTTAAGTGT using an initial denaturation 5 min at 95°C, followed by 5 cycles of 95°C (30 s), 58°C (30 s), 72°C (1 min), followed by 35 cycles of 95°C (30 s), 55°C (30 s), 72°C (1 min), and final extension 5 min at 72°C followed by maintenance/storage at 4°C until sent for sequencing by Sanger sequencing at Azenta Life Sciences (Burlington, MA USA). Sequences were visualized using 4Peaks (RRID: SCR_000015) and the genotype at rs138536707 within the sequence was called manually. Observed genotypes of HG cases were compared to genotypes of 8108 African American individuals in the allele frequency aggregator.^20^

For the non-coding variant rs7761177 upstream of GFRAL, which is in linkage disequilibrium (LD, r^2^ = 0.83) with rs10948901, direct TaqMan genotyping was performed using a proprietary predesigned TaqMan assay (Assay ID C_189464873_10, Thermo Fisher Scientific); the manufacturer-provided VIC/FAM context sequence (GAAGGTTAACACTGGTACAGAAATA[T/C]TTCATAAAAGTTATGAAAACCAGAT) denotes the forward target sequence with the polymorphic site in brackets, where the first base (T) corresponds to the VIC-labeled allele and the second base (C) to the FAM-labeled allele. No imputation was used.

##### GFRAL HG-risk variant rs10948901 genotyping

*GFRAL* variant rs10948901 was genotyped in 40 patients with matched serum samples from *MSC*. PCR was performed with 2× PCR Master Mix (Catalog #K0171, Thermo Fisher Scientific) final volume 20 μL with 20 ng genomic DNA, and 0.25 μM each of forward primer TTGCCAAGGTGTGAGGTCTA and reverse primer ACTTATTGGGCTTTGCCAGC, with an initial denaturation 5 min at 95°C, followed by 5 cycles of 95°C (30 s), 63°C (30 s), 72°C (1 min), followed by 35 cycles of 95°C (30 s), 60°C (30 s), 72°C (1 min), and final extension 5 min at 72°C followed by maintenance/storage at 4°C until sent for sequencing by Sanger sequencing at Azenta Life Sciences (Burlington, MA USA). Sequences were visualized using 4Peaks (RRID: SCR_000015) and the genotype at rs10948901 within the sequence was called manually.

#### In silico mapping of GFRAL mutations to functional domains

To correlate *GFRAL* mutations with clinical phenotype, the cryo-electron microscopy structure of the extracellular dimeric RET/GFRAL/GDF15 complex (Li et al.^19^) (https://www.rcsb.org/structure/6Q2J) was used to map patient-derived *GFRAL* variants onto the respective interaction domains.^19^ As 6Q2J comprises GFRAL residues 1–346 only, the region encompassing Met370 is not resolved. To approximate the GFRAL–RET extracellular interface including this position, we generated AlphaFold3(-Multimer) models of the human GFRAL–RET complex for the wild-type and Met370Thr sequences.^39^ For a qualitative assessment of local dynamics, either model was subjected to a 20 ns explicit-solvent molecular dynamics simulation on the WebGRO for Macromolecular Simulations web server (GROMACS default protocol; https://webgro.uams.edu/).^40^^,^^41^ These *in silico* analyses were used solely to explore a possible structure-function relationship, not as quantitative kinetic measurements.

#### Clinical correlation studies

##### GFRAL HG-risk variant genotyping and HG symptom duration correlation

A common non-coding HG-risk variant mapping upstream of *GFRAL* (rs7761177-T, denoted by T∗)^42^^,^^43^ was analyzed by TaqMan genotyping as described above. Results were correlated with the duration of symptoms in a subset of 125 HG study participants for the whole-exome sequenced cohort that reported on duration of symptoms (resolution before/after 27 weeks).^34^ Odds ratios were calculated using a Fisher’s exact test in an allelic model.

#### Soluble human GFRAL-Fc production

A soluble human GFRAL-Fc fusion protein was produced in Expi293F cells (Thermo Fisher Scientific). The expression construct comprised the codon-optimized extracellular domain of human GFRAL (Ser19–Glu351, UniProt Q6UXV0) fused via an IEGRMD linker to the CH2–CH3 domains of human IgG1 Fc (Pro100–Lys330), cloned into an EF-1α expression plasmid (Twist Bioscience) using NheI/NotI. Expi293F cells were cultured in Expi293 Expression Medium (Thermo Fisher Scientific) at 37°C, 8% CO_2_, 135 rpm and transfected with the EF-1α-GFRAL-Fc plasmid using the Expi293 Expression System (Thermo Fisher Scientific) according to the manufacturer’s protocol. Supernatants were harvested 2–3 days post-transfection by centrifugation and 0.22 μm filtration, supplemented with EDTA-free protease inhibitor, and loaded onto 1 mL HiTrap Protein G HP columns (Cytiva) on an ÄKTA Purifier 10 FPLC system (GE Healthcare) run by the Unicorn software Version 7.0 (Cytiva). After equilibration in 20 mM sodium phosphate, 500 mM NaCl, pH 7.4, bound protein was eluted with 0.1 M glycine, 0.1 M NaCl, pH 2.5. Fractions were immediately neutralized (e.g., 1 M Tris, pH 8–9) and buffer-exchanged into PBS. Purity was assessed by SDS-PAGE, and functional activity was verified by side-by-side comparison to a commercial recombinant human GFRAL-Fc in GDF-15–GFRAL reporter assays.

#### Quantitative GDF-15 and sGFRAL measurements

##### sGFRAL ELISA

Human sGFRAL concentrations in serum or plasma samples were measured using a customized sandwich ELISA. Nunc MaxiSorp™ 96-well ELISA Microplates (ThermoFisher Scientific) were coated overnight at 4°C with 100 μL of 2 μg/mL mouse monoclonal anti-GFRAL antibody (clone 3P10, manufactured by Evitria) diluted in PBS. 3P10 binds GFRAL at the RET-GFRAL interface, with the epitope centered around Arg294.^22^ In this manuscript, the term “sGFRAL” thus always refers to a 3P10-reactive soluble GFRAL ectodomain. Plates were washed 3 times with PBS containing 0.05% Tween®20 (PBST) and blocked with PBS containing 1% BSA (Fraction V, biotin-free, Carl Roth) for 2 h at room temperature (RT) with shaking at 500 rpm. Recombinant mouse GFR alpha-like Fc chimera protein (R&D Systems) was used as the standard. A stock solution was prepared at 40 ng/mL in PBS containing 1% BSA and diluted to 30 ng/mL and 18.2 ng/mL, followed by 1:3 serial dilutions. Blank wells containing PBS with 1% BSA served as negative controls. Serum/plasma samples were analyzed undiluted. Samples, standard and controls were incubated for 2 h at RT with shaking at 500 rpm before plates were washed 3 times with PBST. Captured sGFRAL was detected using 100 μL/well of 50 ng/mL biotinylated sheep polyclonal anti-mouse GFRAL antibody (R&D Systems) diluted in PBS containing 1% BSA for 1 h at RT with shaking at 500 rpm. After washing, plates were incubated with 100 μL/well HRP Streptavidin (BioLegend; 1:1000 in PBS containing 1% BSA). Following a final wash, 100 μL/well of a 1:1 mixture of TMB substrate A and B (TMB Substrate Set, BioLegend) was added. The reaction was stopped with 100 μL of 2N H_2_SO_4_, and absorbance was measured at 450 nm with background correction at 620 nm using a Tecan Sunrise microplate reader (Tecan). Standard curves were fitted using a four-parameter logistic (4PL) regression model in GraphPad Prism 10.3.1 and sample concentrations were interpolated from a standard curve prepared in the same matrix.

Assay validation: The assay was initially validated using in-house produced soluble human GFRAL-Fc (aa19-351). Subsequent quantifications were performed using commercially available recombinant mouse GFR alpha-like Fc chimera protein (R&D Systems), which yielded near-identical results. Cross-validation with spiked samples confirmed the ability to detect sGFRAL in serum or plasma. The intra-assay and inter-assay CVs were <5% and <4%, respectively. Resistance toward up to 3 freeze-thaw cycles and long-term storage stability ≥1 year were confirmed using recombinant human sGFRAL or reanalyzed samples, using the same standards/calibrators. Samples with concentrations below the limit of detection (10 pg/mL) were classified as “undetectable” and assigned a value of 1 in subsequent analyses. To ensure comparability across different plates, all samples (including the IVF and HG samples) were run using the same standards, and comparable results were obtained with calibrators. To ensure reproducibility (while saving precious sample material), technical duplicates were performed for all values. In cases of divergent duplicate measurments (CV > 15%), the measurement was repeated if sufficient sample volume was available.

##### GDF-15 ELISA

Human GDF-15 levels in serum/plasma were determined by ELISA using a modified version of the R&D DuoSet kit (R&D Systems). As the antibody in the original duo set underreports the H202D variant,^44^ we used an in-house-developed human monoclonal anti-GDF-15 antibody for capture.^45^ Despite the use of a different capture antibody, the assay was performed according to the manufacturer’s protocol. Absorbance was measured on a Tecan Sunrise microplate reader (Tecan) at 450 nm with background correction at 620 nm. GraphPad Prism 10.3.1 was used to fit the standard curves to 4 parameters logistic (4PL) model. Quantification of GDF-15 concentrations were based on standard curves prepared in the same matrix. The intra-assay and inter-assay CVs were <6% and <2%, respectively. GDF-15 levels for the HG/morning sickness cohort in Figure 4C were analyzed at the study site using a commercial H202D-insensitive ELISA (Ansh Labs) according to the manufacturer's instructions.

### Quantification and statistical analysis

Standard *t* test for normally distributed data, Pearson’s Chi-Square for proportional differences and pairwise comparison test for identification of specific differences in proportions, with statistical significance defined as alpha *p* < 0.05. Odds ratios, standard errors, and 95% confidence intervals were calculated using the MedCalc Software Ltd. Odds ratio calculator. For the zero-inflated data in Figure 3, where a substantial proportion of values were below the lower limit of detection (LOD = 10 pg/mL), we applied a two-part regression framework to separate (i) the probability of detectable sGFRAL from (ii) the concentration among detectable samples. In the first part, detectability (sGFRAL ≥ LOD vs. < LOD) was modeled by logistic regression with group (HG, NVP, noNVP) and time point (24 vs. 12 weeks) as fixed effects. Odds ratios (ORs) with 95% confidence intervals (CIs) were estimated for HG vs. noNVP, HG vs. NVP, and NVP vs. noNVP, based on either all measurements (12 and 24 weeks combined) or restricted to a single time point (12 or 24 weeks). *p* values for these pairwise group comparisons were multiplicity-adjusted using Tukey’s honestly significant difference (HSD) method. In the second part, restricted to samples with detectable sGFRAL (≥LOD), we analyzed log-transformed sGFRAL concentrations using linear mixed-effects models with group and time point as fixed effects and a random intercept for each woman. Estimated marginal means and pairwise group comparisons were obtained on the log scale and back-transformed to yield fold-changes in sGFRAL between groups with corresponding Tukey HSD–adjusted *p*-values. As for the logistic model, we report both time-specific contrasts at 12 and 24 weeks and integrated contrasts based on all available time points (“all values”). Calculations were performed using RStudio (version 2026.05.0 + 218; R Foundation for Statistical Computing, Vienna, Austria). Correlations between weight loss and sGFRAL levels were assessed using simple linear regression analysis in Prism 10.3.1 (GraphPad). Statistical details of experiments (value of *n*, definition of center, and dispersion and precision measures, value of *p*) are indicated in the respective figure and figure legends.
