# Supplementary material for: GFRAL genetic variations and circulating GFRAL in hyperemesis gravidarum
Source: iScience. 2026 Oct 23;29(11):117975. doi: 10.1016/j.isci.2026.117975 (PMC13720619; doi:10.1016/j.isci.2026.117975)
Supplement: Supplementary file 1 [file mmc1.pdf]

iScience, Volume *29*

Supplemental information

GFRAL genetic variations

and circulating GFRAL in hyperemesis gravidarum

Beatrice Haack, Zainab H. Neemuchwala, Summer DeVera, Kimber W. MacGibbon, Birgitt Fischer, Florian Höschle, Peter A. Fasching, Andrew Housholder, Eszter Vanky, Manuel A. Friese, Marie Bixo, Louise Stiernman, Emelie Ekwurtzel, Malin Nording, Marie-Therese Vinnars, Jörg Wischhusen, and Marlena S. Fejzo

Supplemental Materials

Table of contents

Figure S1: GFRAL Promoter Map with Predicted Binding Sites, related to Figure 4 and discussion.

Table S1: Presence of sGFRAL in HG, NVP and noNVP per trimester, related to Figure 1.

Table S2: HG patients with symptoms resolving between 10-27 weeks vs lasting symptoms, related to results section "*GFRAL* HG-risk variant and prolonged symptom duration".

Table S3: Analysis of circulating sGFRAL in HG, NVP and noNVP cases at 12 and 24 weeks' gestation, related to Figure 3.

Table S4: Nucleotide sequence of hGFRAL(S19-E351) P100-K330-hFc pTwist EF1 Alpha, related to Star Methods.

Figure S1. GFRAL Promoter Map with Predicted Binding Sites, related to Figure 4 and discussion.

(A) **GFRAL Promoter Architecture: Predicted Regulatory Landscape**

Genomic Context

Gene:	GFRAL (GDNF family receptor alpha-like)
Location:	Chromosome 6p12.1 (GRCh38:55,327,469-55,402,493)
Strand:	Forward (+)
Gene Size:	~75 kb
Exons:	9
TSS (Predicted):	~55,327,469 bp (gene start)

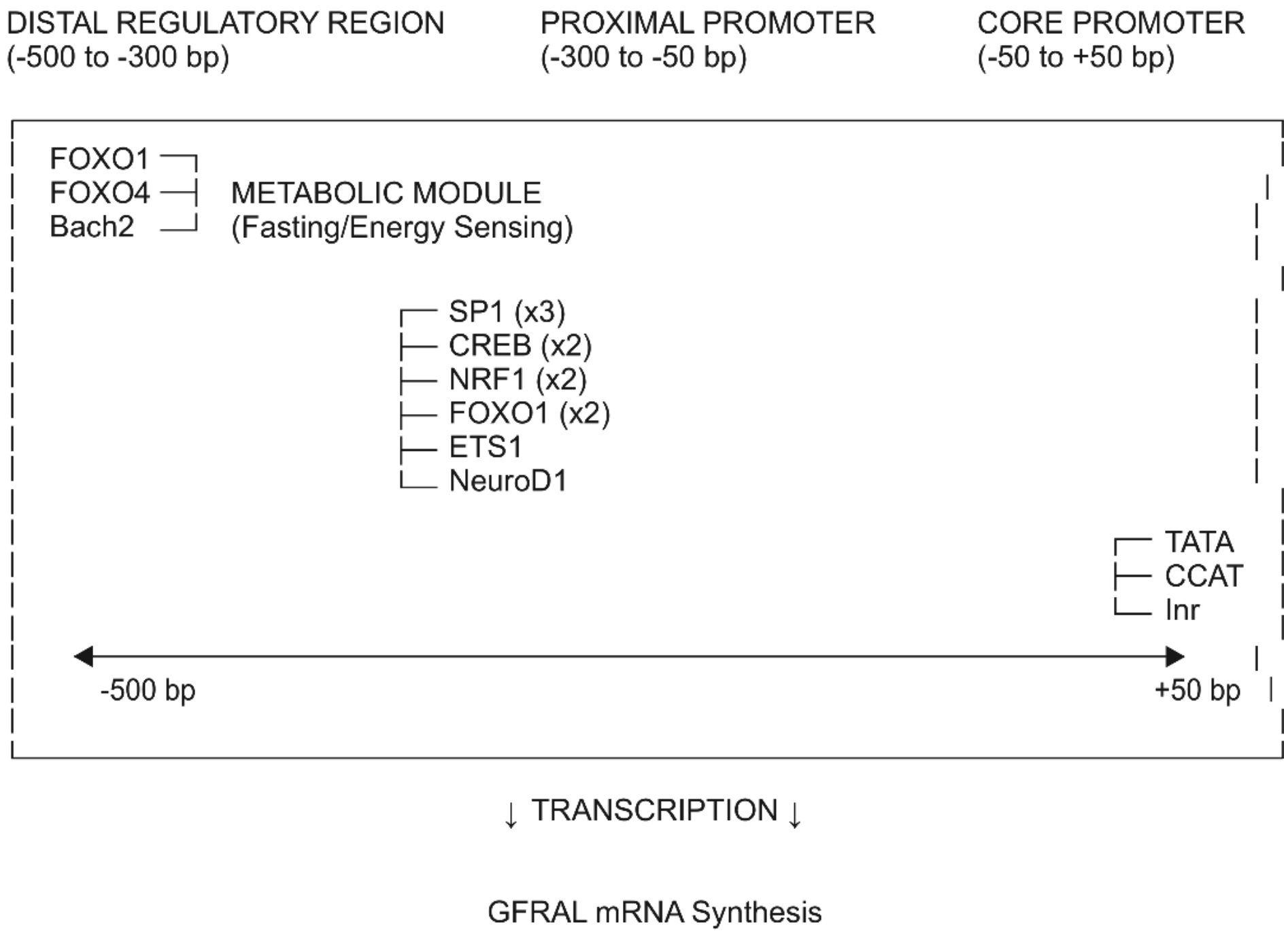

(B) **GFRAL Promoter Map with Predicted Binding Sites**

Position	Element	Consensus Motif	Function	Pregnancy Hormone Effect
**−450 bp**	FOXO1	RYAAACA	Metabolic regulation	↓ Suppressed by PR
**−420 bp**	PRE-1 (Progesterone Response Element)	GGTACAnnnTGTACC	Hormone-mediated repression	↓↓ ACTIVE
**−380 bp**	SP1	GGGCGG	Basal transcription	↓ Reduced by epigenetic silencing
**−350 bp**	FOXO4	RYAAACA	Stress response	↓ Suppressed by PR
**−320 bp**	SP1 (High-confidence)	GGGCGG	Core regulatory site	↓↓ ACTIVE
**−280 bp**	Composite PRE-SP1	GGTACAnnnTGTACC + GGGCGG	Cooperative PR-SP1 binding	↓↓↓ ACTIVE
**−250 bp**	CREB	TGACGTCA	Activity-dependent	↓ Suppressed by epigenetic silencing
**−220 bp**	NRF1	GCGCGC	Metabolic genes	↓ Suppressed by epigenetic silencing
**−180 bp**	PRE-2 (Moderate-affinity)	GGTACAnnnTGTACC	Secondary hormone site	↓ Suppressed by PR
**−150 bp**	CREB	TGACGTCA	Neuronal activity	↓ Suppressed by epigenetic silencing
**−120 bp**	SP1 (High-confidence)	GGGCGG	Core regulatory site	↓↓ ACTIVE
**−85 bp**	NeuroD1	CAGCAG	Brainstem differentiation	↓ Suppressed by epigenetic silencing
**−50 to −30 bp**	TATA Box	TATAAA	Core promoter element	Preserved (basal machinery)
**−25 bp**	CCAAT Box	GGCCAATCT	Core promoter element	Preserved (basal machinery)
**−10 to +10 bp**	Initiator (Inr)	YYANWYY	Transcription start site	Preserved (basal machinery)

GFRAL promoter map with predicted binding sites, including the expected modulation by pregnancy hormones (doi: 10.1093/nar/gkad1059; doi: 10.1093/nar/gkae1080; doi: 10.1007/978-90-481-9069-0_10).

GDNF, glial cell-derived neurotrophic factor; GFRAL, glial cell-derived neurotrophic factor receptor alpha-like; TSS, Transcription Start Site

Table S1. Presence of sGFRAL in HG, NVP and noNVP per trimester, related to figure 1.

PREGNANCY COHORTS	HG sGFRAL	T1	T2	T3	NVP sGFRAL	T1	T2	T3	noNVP sGFRAL	T1	T2	T3
**CGATE COHORT(T1, T2)**	11[a]	7/10	8/11	-	21	13/21	7/21	-	32[a]	9/31	15/32	-
**HG & Morning Sickness Clinic COHORT (T1)**	16	11/16	-	-	0	-	-	-	0	-	-	-
**PMOS COHORT (T1,T3)**	1	1/1	-	1/1	32[a]	12/31	-	11/32	23	10/23	-	11/23
**TOTALS (%)**	-	19/27 (70%)	8/11 (73%)	1/1	-	25/52 (48%)	7/21 (33%)	11/32 (34%)	-	19/54 (35%)	15/32 (47%)	11/23 (48%)

[a] 1 missing from pair

CGATE, Clinical Gravidity Association Trial and Evaluation; GFRAL, Glial cell-derived neurotrophic Factor Receptor Alpha-Like; HG, Hyperemesis Gravidarum; NVP, Nausea and vomiting in pregnancy; sGFRAL, soluble GFRAL; T, trimester

Table S2. HG patients with symptoms resolving between 10-27 weeks vs lasting symptoms, related to results section "*GFRAL* HG-risk variant and prolonged symptom duration".

**Symptoms end**	**</=27 weeks**	**>27 weeks**
T*	32	97
C	44	77

Table S2: HG patients with symptoms resolving between 10-27 weeks vs lasting symptoms. To determine whether a common non-coding HG-risk variant mapping upstream of GFRAL (rs7761177-T, denoted by T*) is associated with symptom duration, resolution of HG was monitored in allele carriers vs. controls.

C, Cytosine; GFRAL, Glial cell-derived neurotrophic Factor Receptor Alpha-Like; HG, Hyperemesis Gravidarum; T, Thymine

Table S3. Analysis of circulating sGFRAL in HG, NVP and noNVP cases at 12 and 24 weeks' gestation, related to Figure 3.

sGFRAL Comparison	N 1	N 2	Mean 1	SS 1	Mean 2	SS 2	t-value	p-value
-	-	-	-	-	-	-	-	-
**12 weeks**	-	-	-	-	-	-	-	-
**HG vs NVP**	10	21	28.12	9723.84	90.78	862303	-0.94	0.35
**HG vs noNVP**	10	31	28.12	9723.84	21.04	49913.4	0.5	0.62
**NVP vs noNVP**	21	31	21.04	49913.4	90.78	862303	1.83	0.07
**HG+NVP vs noNVP**	31	31	70.57	898625	21.04	49913.4	1.55	0.13
**HG vs NVP+noNVP**	10	52	28.12	9723.84	49.21 (54.44)	973107	-0.48	0.64
-	-	-	-	-	-	-	-	-
**24 weeks**	-	-	-	-	-	-	-	-
**HG vs NVP**	11	21	18.03	5103.56	64.37	357551	-1.13	0.27
**HG vs noNVP**	11	32	18.03	5103.56	38.67	99458.2	-1.17	0.25
**NVP vs noNVP**	21	32	64.37	357551	38.67	99458.2	0.97	0.34
**HG+NVP vs noNVP**	32	32	48.44	378157	38.67	99458.2	0.45	0.66
**HG vs NVP+noNVP**	11	53	18.03	5103.56	48.85	465387	-1.07	0.29
-	-	-	-	-	-	-	-	-
**12 vs 24 weeks**	-	-	-	-	-	-	-	-
**HG**	10	11	28.12	9723.84	18.03	5103.56	0.83	0.42
**NVP**	21	21	90.78	862303	64.37	357551	0.49	0.63
**noNVP**	31	32	21.04	49913.4	38.67	99458.2	-1.41	0.16
**All (HG+NVP+noNVP)**	62	64	45.81	986559	43.56	479144	0.12	0.91

GFRAL, glial cell-derived neurotrophic factor receptor alpha-like; HG, Hyperemesis Gravidarum; N, number of cases; NVP, nausea and vomiting in pregnancy; sGFRAL, soluble GFRAL

Table S4. Nucleotide sequence of hGFRAL(S19-E351)_P100-K330-hFc1_pTwist_EF1_Alpha, related to Star Methods.

Nucleotide sequence
AAAGCTTAATGAATTTTGGACTGAGGCTGATTTTCCTTGTGCTTACCCTCAAAGGAGTACAGTGTTCCCAAACTAATAATTGCACGTATCTGAGAGAGCA<br>ATGCCTTCGTGATGCCAATGGGTGTAAACATGCTTGGAGAGTCATGGAAGATGCCTGCAATGATAGCGATCCGGGTGACCCTTGCAAGATGCGCAATA<br>GCTCATACTGTAACCTCAGCATCCAGTACCTCGTCGAAAGCAATTTCCAATTTAAAGAGTGTCTTTGCACTGATGATTTCTATTGTACCGTGAACAAGCTG<br>CTTGGCAAGAAGTGTATAAACAAGTCTGATAACGTTAAAGAGGATAAATTCAAATGGAACCTTACTACTCGCTCACATCATGGATTCAAAGGTATGTGGTC<br>CTGTCTTGAGGTTGCTGAGGCATGTGTGGGCGATGTGGTTTGTAATGCCCAGCTCGCTTCCTACCTTAAAGCCTGCAGCGCGAATGGAAATCCATGTG<br>ATCTGAAACAGTGCCAAGCGGCCATCCGGTTCTTCTATCAAAATATCCCTTTTAACATAGCTCAGATGCTTGCCTTTTGTGACTGTGCTCAATCTGATATT<br>CCTTGTCAGCAGTCTAAAGAGGCCTTGCACTCTAAGACTTGTGCTGTGAACATGGTGCCGCCCCCTACATGCCTGTCAGTGATAAGGTCATGCCAAAAT<br>GATGAATTGTGCCGAAGGCACTATAGAACATTTCAGTCAAAATGCTGGCAGAGAGTGACGCGCAAGTGCCATGAAGATGAGAATTGCATCAGCACACT<br>GAGCAAACAGGACCTCACGTGTTCAGGATCAGATGACTGCAAAGCCGCTTACATCGATATCTTGGGTACCGTCCTCGCAAGTCCAATGTACCTGTAGAAC<br>GATTACACAATCTGAGGAAAGCCTGTGTAAGATTTTCCAGCACATGCTGCATCGCAAATCTTGTTTCAATTATCCAACCCTCTCAAATGTTAAAGGAATGG<br>CCCTCTATACCAGGAAACATGCGAACAAAATCACACTGACTGGTTTTCATAGTCCCTTCAATGGGGAGTCTGGGGGATCCGGGCCCAAAAGCTGTGAC<br>AAAACTCACACATGCCCACCCTGTCCGGCACCGGAATTGCTGGGCGGACCAAGTGTGTTTCTGTTCCCACCCAAACCAAAGGATACCCTCATGATCTC<br>ACGCACACCTGAGGTTACTTGTGTGGTGGTGGATGTCTCCCATGAGGACCCAGAGGTTAAATTCAACTGGTACGTGGATGGAGTGGAAGTTCACAATG<br>CCAAAACCAAGCCTAGAGAGGAACAGTACAATAGTACCTACAGGGTAGTTTCAGTGCTGACAGTGCTGCATCAGGATTGGTTGAATGGTAAGGAATACA<br>AGTGTAAAGTATCTAACAAGGCCCTGCCCGCTCCCATTGAGAAAACCATCTCTAAGGCGAAGGGCCAGCCTCGAGAACCACAGGTTTACACGTTGCCA<br>CCCTCACGAGAGGAAATGACGAAGAACCAGGTCAGTCTCACATGCCTGGTCAAAGGCTTCTACCCGAGCGACATTGCAGTGGAGTGGGAGAGCAATG<br>GGCAGCCGGAGAACAACTACAAAACAACGCCCCCTGTTCTGGATTCAGATGGATCATTCTTTCTCTACTCAAAACTGACAGTTGACAAGAGTCGTTGG<br>CAGCAGGGGAATGTCTTTTCATGCAGTGTAATGCATGAGGCACTGCATAACCACTACACCCAGAAATCCCTTTCACTTAGCCCCGGCAAATGACATCTA<br>GAAT,AAAGCTTAATGAATTTTGGACTGAGGCTGATTTTCCTTGTGCTTACCCTCAAAGGAGTACAGTGTTCCCAAACTAATAATTGCACGTATCTGAGAG<br>AGCAATGCCTTCGTGATGCCAATGGGTGTAAACATGCTTGGAGAGTCATGGAAGATGCCTGCAATGATAGCGATCCGGGTGACCCTTGCAAGATGCGC<br>AATAGCTCATACTGTAACCTCAGCATCCAGTACCTCGTCGAAAGCAATTTCCAATTTAAAGAGTGTCTTTGCACTGATGATTTCTATTGTACCGTGAACAA<br>GCTGCTTGGCAAGAAGTGTATAAACAAGTCTGATAACGTTAAAGAGGATAAATTCAAATGGAACCTTACTACTCGCTCACATCATGGATTCAAAGGTATGT<br>GGTCCTGTCTTGAGGTTGCTGAGGCATGTGTGGGCGATGTGGTTTGTAATGCCCAGCTCGCTTCCTACCTTAAAGCCTGCAGCGCGAATGGAAATCCA<br>TGTGATCTGAAACAGTGCCAAGCGGCCATCCGGTTCTTCTATCAAAATATCCCTTTTAACATAGCTCAGATGCTTGCCTTTTGTGACTGTGCTCAATCTG<br>ATATTCCTTGTCAGCAGTCTAAAGAGGCCTTGCACTCTAAGACTTGTGCTGTGAACATGGTGCCGCCCCCTACATGCCTGTCAGTGATAAGGTCATGCC<br>AAAATGATGAATTGTGCCGAAGGCACTATAGAACATTTCAGTCAAAATGCTGGCAGAGAGTGACGCGCAAGTGCCATGAAGATGAGAATTGCATCAGCA<br>CACTGAGCAAACAGGACCTCACGTGTTCAGGATCAGATGACTGCAAAGCCGCTTACATCGATATCTTGGGTACCGTCCTCGCAAGTCCAATGTACCTGTA<br>GAACGATTACACAATCTGAGGAAAGCCTGTGTAAGATTTTCCAGCACATGCTGCATCGCAAATCTTGTTTCAATTATCCAACCCTCTCAAATGTTAAAGGA<br>ATGGCCCTCTATACCAGGAAACATGCGAACAAAATCACACTGACTGGTTTTCATAGTCCCTTCAATGGGGAGTCTGGGGGATCCGGGCCCAAAAGCTG<br>TGACAAAACTCACACATGCCCACCCTGTCCGGCACCGGAATTGCTGGGCGGACCAAGTGTGTTTCTGTTCCCACCCAAACCAAAGGATACCCTCATGA<br>TCTCACGCACACCTGAGGTTACTTGTGTGGTGGTGGATGTCTCCCATGAGGACCCAGAGGTTAAATTCAACTGGTACGTGGATGGAGTGGAAGTTCAC<br>AATGCCAAAACCAAGCCTAGAGAGGAACAGTACAATAGTACCTACAGGGTAGTTTCAGTGCTGACAGTGCTGCATCAGGATTGGTTGAATGGTAAGGAA<br>TACAAGTGTAAAGTATCTAACAAGGCCCTGCCCGCTCCCATTGAGAAAACCATCTCTAAGGCGAAGGGCCAGCCTCGAGAACCACAGGTTTACACGTT<br>GCCACCCTCACGAGAGGAAATGACGAAGAACCAGGTCAGTCTCACATGCCTGGTCAAAGGCTTCTACCCGAGCGACATTGCAGTGGAGTGGGAGAG<br>CAATGGGCAGCCGGAGAACAACTACAAAACAACGCCCCCTGTTCTGGATTCAGATGGATCATTCTTTCTCTACTCAAAACTGACAGTTGACAAGAGTCG<br>TTGGCAGCAGGGGAATGTCTTTTCATGCAGTGTAATGCATGAGGCACTGCATAACCACTACACCCAGAAATCCCTTTCACTTAGCCCCGGCAAATGACA<br>TCTAGAATGCTAGCTTGACTGACTGAGATACAGCGTACCTTCAGCTCACAGACATGATAAGATACATTGATGAGTTTGGACAAACCACAACTAGAATGCA<br>GTGAAAAAAATGCTTTATTTGTGAAATTTGTGATGCTATTGCTTTATTTGTAACCATTATAAGCTGCAATAAACAAGTTAACAACAACAATTGCATTCATTTTA<br>TGTTTCAGGTTCAGGGGGAGGTGTGGGAGGTTTTTTAAAGCAAGTAAAACCTCTACAAATGTGGTATTGGCCCATCTCTATCGGTATCGTAGCATAACCC<br>CTTGGGGCCTCTAAACGGGTCTTGAGGGGTTTTTTGTGCCCCTCGGGCCGGATTGCTATCTACCGGCATTGGCGCAGAAAAAAATGCCTGATGCGAC<br>GCTGCGCGTCTTATACTCCCACATATGCCAGATTCAGCAACGGATACGGCTTCCCCAACTTGCCCACTTCCATACGTGTCCTCCTTACCAGAAATTTATC<br>CTTAAGGTCGTCAGCTATCCTGCAGGCGATCTCTCGATTTCGATCAAGACATTCCTTTAATGGTCTTTTCTGGACACCACTAGGGGTCAGAAGTAGTTCA<br>TCAAACTTTCTTCCCTCCCTAATCTCATTGGTTACCTTGGGCTATCGAAACTTAATTAACCAGTCAAGTCAGCTACTTGGCGAGATCGACTTGTCTGGGT<br>TTCGACTACGCTCAGAATTGCGTCAGTCAAGTTCGATCTGGTCCTTGCTATTGCACCCGTTCTCCGATTACGAGTTTCATTTAAATCATGTGAGCAAAAG<br>GCCAGCAAAAGGCCAGGAACCGTAAAAAGGCCGCGTTGCTGGCGTTTTTCCATAGGCTCCGCCCCCCTGACGAGCATCACAAAAATCGACGCTCAAG<br>TCAGAGGTGGCGAAACCCGACAGGACTATAAAGATACCAGGCGTTTCCCCCTGGAAGCTCCCTCGTGCGCTCTCCTGTTCCGACCCTGCCGCTTACC<br>GGATACCTGTCCGCCTTTCTCCCTTCGGGAAGCGTGGCGCTTTCTCATAGCTCACGCTGTAGGTATCTCAGTTCGGTGTAGGTCGTTCGCTCCAAGCT<br>GGGCTGTGTGCACGAACCCCCCGTTCAGCCCGACCGCTGCGCCTTATCCGGTAACTATCGTCTTGAGTCCAACCCGGTAAGACACGACTTATCGCCA<br>CTGGCAGCAGCCACTGGTAACAGGATTAGCAGAGCGAGGTATGTAGGCGGTGCTACAGAGTTCTTGAAGTGGTGGCCTAACTACGGCTACACTAGAA<br>GAACAGTATTTGGTATCTGCGCTCTGCTGAAGCCAGTTACCTTCGGAAAAAGAGTTGGTAGCTCTTGATCCGGCAAACAAACCACCGCTGGTAGCGGT<br>GGTTTTTTTGTTTGCAAGCAGCAGATTACGCGCAGAAAAAAAGGATCTCAAGAAGATCCTTTGATCTTTTCTACGGGGTCTGACGCTCAGTGGAACGAA<br>AACTCACGTTAAGGGATTTTGGTCATGAGATTATCAAAAAGGATCTTCACCTAGATCCTTTTAAATTAAAAATGAAGTTTTAAATCAATCTAAAGTATATATG<br>AGTAAACTTGGTCTGACAGTTACCAATGCTTAATCAGTGAGGCACCTATCTCAGCGATCTGTCTATTTCGTTCATCCATAGTTGCATTTAAATTTCCGAACT<br>CTCCAAGGCCCTCGTCGGAAAATCTTCAAACCTTTCGTCCGATCCATCTTGCAGGCTACCTCTCGAACGAACTATCGCAAGTCTCTTGGCCGGCCTTG<br>CGCCTTGGCTATTGCTTGGCAGCGCCTATCGCCAGGTATTACTCCAATCCCGAATATCCGAGATCGGGATCACCCGAGAGAAGTTCAACCTACATCCTC<br>AATCCCGATCTATCCGAGATCCGAGGAATATCGAAATCGGGGCGCGCCTGGTGTACCGAGAACGATCCTCTCAGTGCGAGTCTCGACGATCCATATCG<br>TTGCTTGGCAGTCAGCCAGTCGGAATCCAGCTTGGGACCCAGGAAGTCCAATCGTCAGATATTGTACTCAAGCCTGGTCACGGCAGCGTACCGATCTG<br>TTTAAACCTAGATATTGATAGTCTGATCGGTCAACGTATAATCGAGTCCTAGCTTTTGCAAACATCTATCAAGAGACAGGATCAGCAGGAGGCTTTCGCAT<br>GAGTATTCAACATTTCCGTGTCGCCCTTATTCCCTTTTTTGCGGCATTTTGCCTTCCTGTTTTTGCTCACCCAGAAACGCTGGTGAAAGTAAAAGATGCT<br>GAAGATCAGTTGGGTGCGCGAGTGGGTTACATCGAACTGGATCTCAACAGCGGTAAGATCCTTGAGAGTTTTCGCCCCGAAGAACGCTTTCCAATGAT<br>GAGCACTTTTAAAGTTCTGCTATGTGGCGCGGTATTATCCCGTATTGACGCCGGGCAAGAGCAACTCGGTCGCCGCATACACTATTCTCAGAATGACTT<br>GGTTGAGTATTCACCAGTCACAGAAAAGCATCTTACGGATGGCATGACAGTAAGAGAATTATGCAGTGCTGCCATAACCATGAGTGATAACACTGCGGC<br>CAACTTACTTCTGACAACGATTGGAGGACCGAAGGAGCTAACCGCTTTTTTGCACAACATGGGGGATCATGTAACTCGCCTTGATCGTTGGGAACCGG<br>AGCTGAATGAAGCCATACCAAACGACGAGCGTGACACCACGATGCCTGTAGCAATGGCAACAACCTTGCGTAAACTATTAACTGGCGAACTACTTACTC<br>TAGCTTCCCGGCAACAGTTGATAGACTGGATGGAGGCGGATAAAGTTGCAGGACCACTTCTGCGCTCGGCCCTTCCGGCTGGCTGGTTTATTGCTGAT<br>AAATCTGGAGCCGGTGAGCGTGGGTCTCGCGGTATCATTGCAGCACTGGGGCCAGATGGTAAGCCCTCCCGTATCGTAGTTATCTACACGACGGGGA<br>GTCAGGCAACTATGGATGAACGAAATAGACAGATCGCTGAGATAGGTGCCTCACTGATTAAGCATTGGTAACCGATTCTAGGTGCATTGGCGCAGAAAA<br>AAATGCCTGATGCGACGCTGCGCGTCTTATACTCCCACATATGCCAGATTCAGCAACGGATACGGCTTCCCCAACTTGCCCACTTCCATACGTGTCCTC<br>CTTACCAGAAATTTATCCTTAAGATCCCGAATCGTTTAAACTCGACTCTGGCTCTATCGAATCTCCGTCGTTTCGAGCTTACGCGAACAGCCGTGGCGCT<br>CATTTGCTCGTCGGGCATCGAATCTCGTCAGCTATCGTCAGCTTACCTTTTTGGCAGCGATCGCGGCTCCCGACATCTTGGACCATTAGCTCCACAGGT<br>ATCTTCTTCCCTCTAGTGGTCATAACAGCAGCTTCAGCTACCTCTCAATTCAAAAAACCCCTCAAGACCCGTTTAGAGGCCCCAAGGGGTTATGCTATCA<br>ATCGTTGCGTTACACACACAAAAAACCAACACACATCCATCTTCGATGGATAGCGATTTTATTATCTAACTGCTGATCGAGTGTAGCCAGATCTCGTGAG<br>GCTCCGGTGCCCGTCAGTGGGCAGAGCGCACATCGCCCACAGTCCCCGAGAAGTTGGGGGGAGGGGTCGGCAATTGAACCGGTGCCTAGAGAAGG<br>TGGCGCGGGGTAAACTGGGAAAGTGATGTCGTGTACTGGCTCCGCCTTTTTCCCGAGGGTGGGGGAGAACCGTATATAAGTGCACTAGTCGCCGTGA<br>ACGTTCTTTTTCGCAACGGGTTTGCCGCCAGAACACAGGTAAGTGCCGTGTGTGGTTCCCGCGGGCCTGGCCTCTTTACGGGTTATGGCCCTTGCGT<br>GCCTTGAATTACTTCCACCTGGCTGCAGTACGTGATTCTTGATCCCGAGCTTCGGGTTGGAAGTGGGTGGGAGAGTTCGTGGCCTTGCGCTTAAGGA<br>GCCCCTTCGCCTCGTGCTTGAGTTGTGGCCTGGCCTGGGCGCTGGGGCCGCCGCGTGCGAATCTGGTGGCACCTTCGCGCCTGTCTCGCTGCTTT<br>CGATAAGTCTCTAGCCATTTAAAATTTTTGATGACCTGCTGCGACGCTTTTTTTCTGGCAAGATAGTCTTGTAAATGCGGGCCAAGATCAGCACACTGGT<br>ATTTCGGTTTTTGGGGCCGCGGGCGGCGACGGGGCCCGTGCGTCCCAGCGCACATGTTCGGCGAGGCGGGGCCTGCGAGCGCGGCCACCGAGAA<br>TCGGACGGGGGTAGTCTCAAGCTGCCCGGCCTGCTCTGGTGCCTGGCCTCGCGCCGCCGTGTATCGCCCCGCCCTGGGCGGCAAGGCTGGCCCG<br>GTCGGCACCAGTTGCGTGAGCGGAAAGATGGCCGCTTCCCGGCCCTGCTGCAGGGAGCACAAAATGGAGGACGCGGCGCTCGGGAGAGCGGGCG<br>GGTGAGTCACCCACACAAAGGAAAAGGGCCTTTCCGTCCTCAGCCGTCGCTTCATGTGACTCCACGGAGTACCGGGCGCCGTCCAGGCACCTCGAT<br>TAGTTCTCCAGCTTTTGGAGTACGTCGTCTTTAGGTTGGGGGGAGGGGTTTTATGCGATGGAGTTTCCCCACACTGAGTGGGTGGAGACTGAAGTTAG<br>GCCAGCTTGGCACTTGATGTAATTCTCCTTGGAATTTGCCCTTTTTGAGTTTGGATCTTGGTTCATTCTCAAGCCTCAGACAGTGGTTCAAAGTTTTTTT<br>CTTCCATTTCAGGTGTCGTGAAAACTACCCCTAAAAGCCAAAAGATCTTTGTCGATCCTACCATCCACTCGACACACCCGCCAGCGGCCGC
